# Engaging a Community for Rare Genetic Disease: Best Practices and Education From Individual Crowdfunding Campaigns

**DOI:** 10.2196/ijmr.7176

**Published:** 2018-02-05

**Authors:** Romina Alicia Ortiz, Steven Witte, Arvin Gouw, Ana Sanfilippo, Richard Tsai, Danielle Fumagalli, Christine Yu, Karla Lant, Nicole Lipitz, Jennifer Shepphird, Fidelia B Alvina, Jimmy Cheng-Ho Lin

**Affiliations:** ^1^ Rare Genomics Institute Downey, CA United States

**Keywords:** surveys and questionnaires, patient empowerment, fund raising, benchmarking, internet, genetic testing, molecular diagnostic techniques, social planning, health education

## Abstract

**Background:**

Genetic sequencing is critically important to diagnostic health care efforts in the United States today, yet it is still inaccessible to many. Meanwhile, the internet and social networking have made crowdfunding a realistic avenue for individuals and groups hoping to fund medical and research causes, including patients in need of whole exome genetic sequencing (WES).

**Objective:**

Amplify Hope is an educational program designed to investigate what factors affect the success of medical crowdfunding campaigns. We conducted a needs assessment, a series of 25 interviews concerning crowdfunding, and provided training on best practices identified through our assessment for 11 individuals hoping to run their medical crowdfunding campaigns to raise money for patients to access trio WES to identify the mutated proteins that caused their apparent inherited disease.

**Methods:**

The crowdfunding education was given in a 30-day training period with resources such as webinars, fact sheets and a crowdfunding training guide emailed to each participant. All campaigns were launched on the same date and were given 30 days to raise the same goal amount of US $5000. Reviewing the 4 crowdfunding campaigns that raised the goal amount within the 30-day period, we sought to identify features that made the 4 crowdfunding campaigns successful. In addition, we sought to assess which factors the resulting 75 donors report as influencing their decision to donate to a campaign. Finally, we investigated whether crowdfunding campaigns for exome sequencing had an impact on increasing applicant’s and donors’ knowledge of genomics.

**Results:**

Of the 86 study inquiries, 11 participants submitted the required forms and launched their crowdfunding campaigns. A total of 4 of the 11 campaigns raised their goal amounts within 30 days.

**Conclusions:**

We found that social media played an important role in all campaigns. Specifically, a strong social media network, an active outreach process to networks, as well as engagement within the study all correlated with a higher success rate. Amplify Hope donors were more likely to support projects that were near their fundraising goals, and they found video far more effective for learning about genomics than any other medium.

## Introduction

### Genetics in Society

DNA sequencing is at the forefront of many cutting-edge research and treatment programs, especially for genetic diseases. The diagnostic yield of whole exome genetic sequencing (WES) is reported to be 25% to 34% for children with undiagnosed genetic disease [1-3]. WES reduces the amount of time needed to reach a diagnosis compared with the current standard of care [1], and it has the advantage of being able to identify novel mutations [2]. Genetic sequencing also plays a central role in the implementation of personalized medicine, an increasingly important priority in our society. The Precision Medicine Initiative introduced by President Obama in 2015 aims to accelerate research efforts by enhancing data access and collaborations between researchers, doctors, and patients; a key component of the project includes genetic sequencing. In another nationwide initiative, the PrecisionFDA Consistency Challenge works to improve personalized care by achieving more consistent results in genetic tests.

Advances in genetic sequencing technology have substantially reduced its cost, making it more accessible to patients and researchers. Less than 20 years ago, sequencing the entire human genome cost billions of dollars, but today, the cost to sequence the most relevant parts of the genome and provide information as a clinical test option is in the thousands [4]. WES has become the gold standard of advanced genetic testing to determine the underlying cause of a patient’s undiagnosed illness. Majority of inherited diseases identified to date result from mutated proteins [5], and WES is the sequencing of the complete protein-coding regions (exome) of the human genome [6].

Although the cost to sequence a genome has been substantially reduced, it is still unaffordable for many patients because it is not routinely covered by health insurance. These out-of-pocket expenses are a financial strain for the patients that need testing for diagnosis. Looking just at financial strain for a typical person, a 2011 poll from the National Bureau of Economic Research found that nearly half of the respondents stated that if they were given a 30-day time frame, they would be unable to produce an extra US $2000 [7]. Beyond overtaxed household budgets, families dealing with undiagnosed or rare diseases incur countless unexpected health care costs that create tremendous financial burdens. These families shoulder the staggering health care expenses accumulated over years of seeking treatment.

### Engaging a Community

The increasing reach of the internet and social networking has created a new channel for fundraising through internet-based crowdfunding, which asks many people via websites and social media to donate money and provide support to an individual project or a campaign. Organizers of crowdfunding campaigns use their online connections and offline activities to promote their campaigns. Unlike traditional funding that comes from a few large investors or campaigns that ask for a specific donation amount, crowdfunding allows anyone to donate any amount. In light of the diminishing funding for research grants, crowdfunding enables individuals and groups worldwide to bridge the funding gap and support medical and research causes [8]. Crowdfunding may help support patients in need of DNA sequencing by providing access to a test that might uncover the cause of their disease and potentially lead to a viable clinical treatment.

Although previous research has examined crowdfunding as a novel means for raising money and support, few studies have focused on the factors that improve crowdfunding outcomes. Our literature search identified just one study that focused exclusively on crowdfunding for personal medical expenses. Although several studies discuss the possibilities of crowdfunding for larger medical research projects that are not able to secure funding through traditional sources, the studies did not examine factors that contribute to successful campaigns. Burtch and Chan [9] found evidence that crowdfunding campaigns were correlated with a reduction in medical bankruptcies in the United States, concluding that crowdfunding helped reduce the rate of bankruptcy due to medical expenses by 3.7% in 2014. However, they do not go further beyond establishing this link to examine the factors responsible for the success of crowdfunding campaigns or the donors’ motivations for giving.

Two key drawbacks of the existing body of research limit its relevance to the funding of personal medical expenses. First, most studies have evaluated platforms that offer donors a return, such as a final product from a Kickstarter campaign, or interest on loans from the crowdfunding platform Prosper. Purely charitable crowdfunding projects have not been studied. Second, many of the platforms analyzed adopted an “all-or-nothing” policy, whereby if the goal goes unmet, the donations are returned. This feature along with the presence (or absence) of a reward almost certainly influences some individual decisions to contribute. Findings based on postcampaign surveys of donors are also limited.

With the gap in financial support for undiagnosed individuals, Rare Genomics (RG) began allowing patients and their families to create crowdfunding projects since 2012. These campaigns were on the RG website and set to raise the funds needed for clinical WES with partnering RG laboratories. Early campaigns varied in success, ranging from hours to days to months. Amplify Hope was then designed to investigate existing strategies for crowdfunding, educate participants preparing to crowdfund, and determine the effectiveness of crowdfunding strategies used as well as donor engagement.

## Methods

### Study Design

RG conducted the study in 5 phases, including a needs assessment to identify successful crowdfunding strategies, participant recruitment, a 30-day crowdfunding training period, a 30-day online crowdfunding period, and follow-up surveys given to participants and donors. Our research sought to (1) provide demographic information on the donor population; (2) identify common factors among successful medical crowdfunding campaigns; (3) identify factors that influenced people to donate, as reported by donors; and (4) describe the impact crowdfunding campaigns had on donors’ self-reported knowledge of genomics.

CrowdRise, Indiegogo Life, and YouCaring were the crowdfunding platform partners. They provided the following information to be used for analytical purposes: the average donation to a fundraiser, unique page visits, conversion rates, and how many people had visited each page. We utilized 3 different crowdfunding sites to see if there was a measurable difference in terms of successful campaigns. Baylor Miraca Genetics Laboratories and Ambry Genetics were the sequencing partners in the study. They provided WES for all patients with successful crowdfunding campaigns.

### Needs Assessment

A needs assessment was conducted to elucidate current crowdfunding practices and their effectiveness. The first phase consisted of 25 phone interviews with crowdfunding experts and founders of crowdfunding platforms as well as individuals who had run successful crowdfunding campaigns for personal medical care or scientific research projects. Ethan Austin, Breanna DiGiammarino, Adam Griff, Annette Hayswirth, Elizabeth Iorns, Nick Karolidis, Denny Luan, Molly Lindquist, Andrea Lo, Luke Miner, Jamie McDonald, Sandip Sekhon, Nick Sireau, Devin Thorpe, and Rob Wu are founders of crowdfunding platforms that shared their expertise. Sam De Brouwer, Zsuzsanna Darvai, Ignacio Garcia, Kimmie Ng, Glenn O’Neill, Susanne Shaw, and Jeneva Stone shared their insights about their own crowdfunding campaigns. The interviews were conducted for an average length of 30 min. The purpose of all interviews was to establish crowdfunding best practices, elicit recommendations, and develop materials for the training program phase of the study.

### Participant Population

As the crowdfunding efforts were aimed to raise money needed for WES, inclusion in the study was dependent on 2 factors: that the participant had the desire to actively participate in the study to raise funds online, and that the participant intended to raise money for a patient that had a certified physician’s request for WES.

A total of 13,542 recruitment emails were sent from May 20, 2015, to July 28, 2015, to rare disease advocacy groups and genetic counselors to inform them of the study opportunity for undiagnosed patients. Participants completed an interest form (Multimedia Appendix 1) asking who the intended sequencing was for, whether there was a physician referral, as well as their level of knowledge on WES and crowdfunding. To participants that indicated they had physician support, a Getting Started Worksheet (Multimedia Appendix 2) was sent allowing for a more comprehensive investigation into the patient and participant. The Getting Started Worksheet was designed to determine social media and community presence and activity. Participants who completed both forms were then asked to review a consent form (Multimedia Appendix 3) to enter the study, indicating they would participate in the training, launch their campaign, and provide campaign evaluation. All participants provided contact information for the patient’s referring physician, and referring physicians confirmed the WES order by submission of a signed doctor’s note (Multimedia Appendix 4).

The participants were randomly assigned to one of three crowdfunding sites. Participants in the study were given a 30 day training program. Educational resources on crowdfunding were emailed every week including webinars, fact sheets and a crowdfunding training guide. During the 30 day training program, participants received an eBook on crowdfunding best practices, educational worksheets on both exome sequencing and crowdfunding each week by email and were invited to attend webinars. The training included recommendations on engaging their network of contacts through email and phone calls, team building, creating a campaign video, and social media messaging. At the same time, participants received challenges designed to help them prepare for the campaigns. Incentives were included in the challenges. One challenge awarded prizes of US $200, US $100, and US $50 to the top 3 campaigns that raised the most money on the launch day. Another challenge awarded US $50 to every campaign that completed the campaign-page story by the deadline set before the campaign launch.

The fundraising goal for all participants was set to US $5000, the cost of a trio WES with partnering laboratories Ambry Genetics and Baylor Miraca Laboratories. The time frame was 30 days, with each participant randomly assigned to one of the 3 different crowdfunding sites. Challenge events after the campaign launch provided campaign organizers opportunities to engage with their networks by offering incentives to donate and share. A week-long challenge offered a first prize of US $200, a second prize of US $100, and a third prize of US $50 to the campaigns with the most new donors during the challenge week. A similar challenge offered prize awards for the campaigns with the most social shares for the week. If participants did not achieve their goals after 30 days, the campaigns were extended an additional 60 days. Overall, 2 participants exceeded the goal in 30 days and 4 met the goal within 60 days.

### Effectiveness of Crowdfunding Strategies

To evaluate the effectiveness of the different crowdfunding strategies, the following metrics were assessed: time to complete fundraising, communication engagement with the participants, number of donors, average donation amount and donor education on crowdfunding and sequencing. To gauge communication engagement, we gave communication points for the following two instances: (1) when participants responded to our email or call communications about the study and (2) when participants initiated their own communication to us regarding the study. We subtracted communication points when a participant did not respond to our emails or calls regarding the study. We also noted when these communications had a quick response time (within the same day) and a long response time (over 3 days).

To examine whom the donors were and what factors contributed to their decision to donate to a campaign, demographic analyses of the donors were done utilizing surveys. After a campaign donation was received, donors were invited to respond to an anonymous survey asking about their demographics, such as sex, age range, and educational level, as well as gauging their self-reported baseline knowledge of genomics. Additionally, they were asked about the impact, if any, of the educational materials provided on their knowledge of genomics. They were asked what specifically they read of the information made available to them about genomics. They had the ability to access information from the campaign page and were asked what material did you receive, read, or watch from the campaign (check all that apply). The choices were read the campaign summary, looked at other similar campaigns, watched the TedxMidAtlantic video, read Genomics 101, read eBook(s), and other (please specify).

### Education Through Crowdfunding

To determine the baseline self-reported knowledge of the participants, they were asked to rate their crowdfunding and sequencing knowledge from the initial interest form (SF1) from a scale of 0-10. Participants had the ability to access educational information from the campaign and were asked what material they did receive/read/watch from the campaign (check all that apply). The choices were as follows: (1) read the campaign summary, (2) looked at other similar campaigns, (3) watched the TedxMidAtlantic video, (4) read Genomics 101 pamphlet, (5) read eBook(s), and (6) other (please specify). We used postcampaign surveys to determine if the crowdfunding campaigns increased participants’ and donors’ knowledge of genomics or increased interest in learning about it.

## Results

### Needs Assessment

The takeaways from individual phone interviews with 25 crowdfunding platform founders and people who led successful campaigns were prepared in the document “Crowdfunding Best Practices” (Multimedia Appendix 5) and were used to train the participants in the study. Best practices identified include having a “pregame plan” before the start date of the campaign where you set a clear measurable goal, create a message and strategy for your target community, arrange initial commitments, and create media for the campaign including high-quality photos, a video, and written materials. Best practices during the campaign include holding a launch party, providing regular and detailed updates, use of social media daily and media contacts, linking of offline fundraising to the online work, and eliciting feedback and acting on it.

### Participant Population

**Figure 1:**
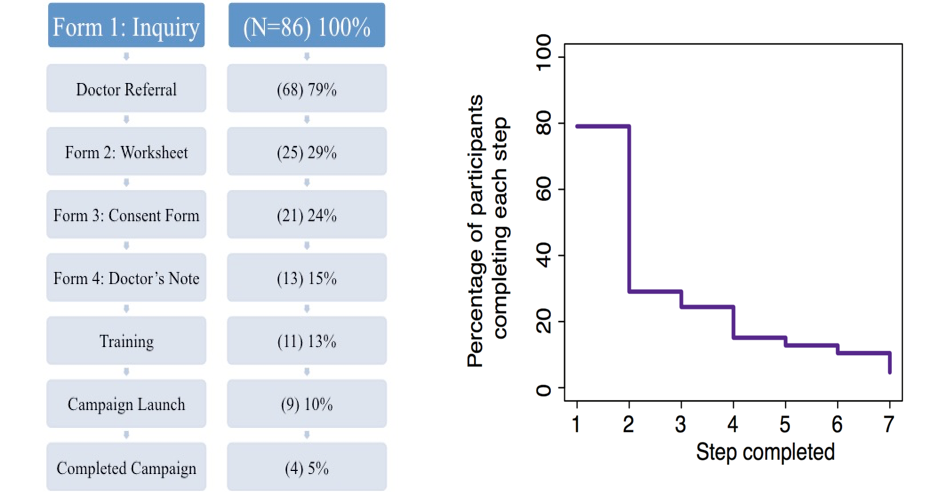
Of the 86 people that expressed interest in the study, 4 people (5%) successfully completed all subsequent steps, including the funding campaign (step 7).

From the 86 completed inquiries, 68 applicants (79%) affirmed they had doctor referrals for the test (Figure 1). From the 68 that appeared to have medical support to request the test, 25 (less than 40%) completed Form 2 and 84% of those participants submitted signed consent forms (Multimedia Appendix 3) for the study, dropping the participant number to 21. The study team received signed doctor’s notes for 13 of the 21 participants. The 8 referring physicians who did not submit doctor’s notes were either unresponsive or stated that they did not recommend WES. All 13 cases with completed forms were invited to enter the study for training. One of the cases became unresponsive and did not enter training. Of the 12 that participated in the training, one participant dropped out during training due to lack of ability to commit time to the effort.

The principal sources of applicants were the patients themselves (38 out of 86) and parents of the patients (38 out of 86). A total of 65 (76%) of the 86 that applied were females and 21 (24%) were males. The ages were concentrated between 40 and 49 years. The second largest group was between 30 and 39 years. A total of 66 of the 86 (76%) applicants had a bachelor’s degree or higher, but at the time of the study, 52 out of the 86 (60%) participants were either unemployed or disabled.

### Effectiveness of Crowdfunding Strategies

From the 11 participants who completed training for the study, one secured complete sponsorship for their campaign during the training program before the launch, and one participant won a free WES test with first place in one of the study’s training challenges. In total, 9 crowdfunding campaigns were launched, and 4 campaigns reached their funding goals of US $5500.

One campaign was funded during preparation of the launch, and the other 3 campaigns that did launch each utilized a different crowdfunding platform—CrowdRise, Indiegogo Life, and YouCaring. Thus, all of the crowdfunding platforms had successful campaigns represented in our study.

We observed a few overlapping characteristics among the campaigns that achieved complete funding by the end of the study period in both internal engagement with the study coordinators as well as external engagement with social networks before, during, and at the end of the study. Higher levels of engagement were observed throughout the training before the start of the crowdfunding campaigns as compared with those that did not achieve their campaign goals. This included communication engagement as well as completion of study and campaign tasks.

By the measure of internal study engagement, all successful crowdfunding campaigns had 4 or more total communication engagement points, whereas majority of the unsuccessful crowdfunding campaigns had 3 or less points total. Of note is participant #8 that had 12 engagement points total and still was unsuccessful. Interestingly, none of the successful crowdfunding campaigns had an instance where the participants did not respond to either email or phone communication from the study organizer (no responses). All successful campaigns had points for self-initiated engagement, and all but one unsuccessful campaign also had points for self-initiated engagement. However, successful campaigns had an average of 2.75 points, whereas unsuccessful campaigns had an average of 1.67 points. Moreover, successful campaigns had an average of 4 points for a quick response time of 1 day or less. Unsuccessful campaigns only had an average of 2.5 points. From both successful and unsuccessful campaigns, the number of instances with slow response times were insignificant at less than 0.5 points average for both.

Most participants used social media as a tool to engage their social network to raise funds, and most were already well connected through many platforms: 8 of the 9 reported they used Facebook (3 of the 9 had 500 or more Facebook friends), 6 of the 9 used Twitter, and 5 of the 9 used Google + and LinkedIn. In addition, many regularly shared updates about their rare disease journey on (8 of the 9) Facebook and Twitter (4 of the 9). Once the 30-day campaigns launched online, a total of 334 social media messages were shared on Twitter, Facebook, LinkedIn, and Google +. Of the 334 total social media posts, 217 (64.9%) were shared, mostly on Facebook and Twitter. Embedded links in the shared posts directed the user to an individual participant’s crowdfunding site or to the Amplify Hope website. The click rate for embedded links was 70.0% (152/217) of shared messages, 67.8% (103/152) from Twitter posts and 32.2% (49/152) from Facebook posts. Of the total shared posts, 58.5% (127/217) garnered some form of interaction (eg, they were retweeted, liked, favorited, or shared) at least once (53.5% [68/127 on Twitter and 47% [59/127] on Facebook).

**Figure 2:**
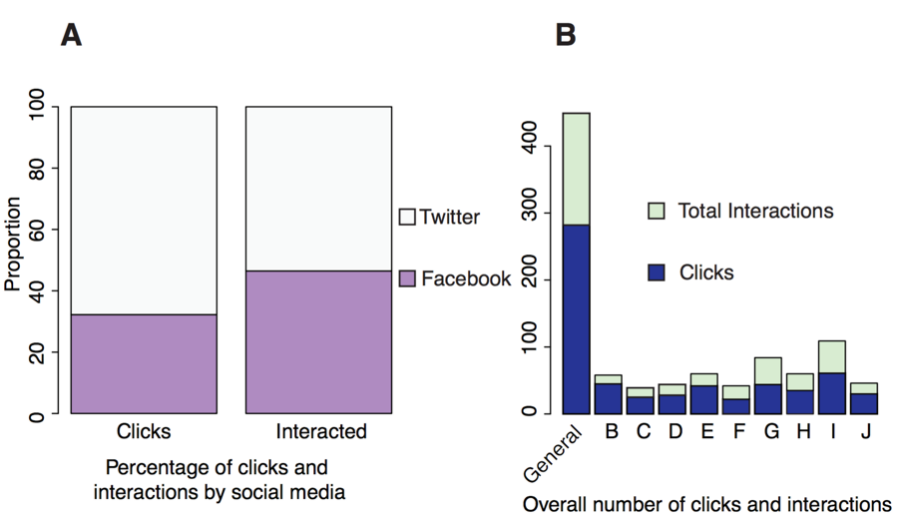
Analysis of social media messages. (A) Breakdown of clicks and interactions of social media posts made via Twitter and Facebook. (B) Analysis of engagement of social media posts for individual campaigns. “General” refers to generic messages meant to promote user participation, and “B-J” refers to individual campaigns, which have been anonymized.

Messages promoting the Amplify Hope campaign through social media were divided into the following 2 categories: (1) general campaign messages to promote and encourage user participation and (2) campaign-specific messages tailored to drive traffic to an individual’s campaign. Campaign-specific messages garnered slightly higher total click rates and interaction rates than did general messages on both Twitter (52% vs 44%) and Facebook (55% vs 44%), as shown in Figure 2. Similarly, individual-specific messaging appeared to be somewhat more effective than general messaging in reaching audiences on social media.

Separately, we sought to get a comprehensive understanding of characteristics and strategies used by the successful crowdfunding campaigns. All the 3 successful campaigns were organized by mothers of the patient in need of exome sequencing. In one of the cases, the mother completed the training program. She reported reading all the materials provided and engaged with the study coordinators by asking self-initiated questions throughout the process. She completed all aspects of the precampaign work, reaching out to her network of friends and family to inform them of her upcoming campaign, she wrote a campaign story on the CrowdRise crowdfunding platform, and included 5 photos and a video as recommended. She held a launch party the day the campaign went live online. The campaign won the first place in our challenge of the week for the most new donors and second place in the challenge for the most social shares. Her top source of messaging was Facebook, and during the 30-day online study period, the campaign had 1208 unique user sessions and a total of 74 donors. The campaign raised US $5680, exceeding the US $5500 goal. In the second case, the campaign was conducted from Tanzania using the YouCaring platform. Here too, the campaign organizer participated fully in the 30-day training and reported reviewing all the material provided. Of note, she had some delays in completing the study homework tasks as she had a child in the hospital. However, despite this, she completed the recommended work before launching her 30-day online campaign. She created a campaign story on YouCaring’s platform, which included 7 photos and 1 video with words embedded in the video. She requested contributions at the end of the video and thanked supporters. Similar to the first case, the campaign won challenges. This campaign won first place for the most social shares in 1 week and second place for the most new donors in 1 week. The campaign had 2394 unique users and 3589 total sessions. The campaign exceeded the goal of US $5500 by raising US $5844 with 47 donors. One campaign received full funding during the training program. The campaign organizer was an adult patient in need of exome sequencing. During the training program, he was fully engaged in the training, conducting recommended outreach by email and phone to his network of friends, family, and contacts. He requested feedback on his campaign draft story from his network as well as the study team. During his outreach, one donor agreed to pay the entire cost of exome sequencing, and thus, he was fully funded for US $5500 before the online campaign period of the study.

**Figure 3:**
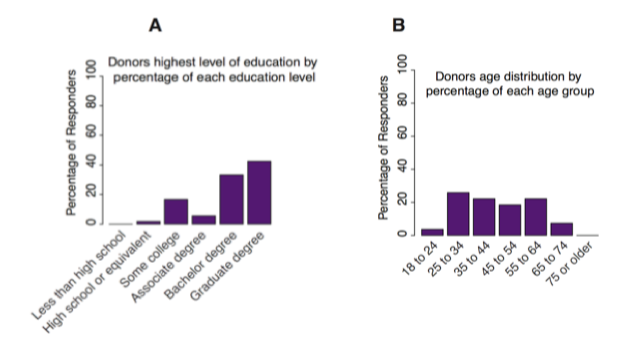
Donor demographic information. (A) Education level of donors. (B) Age of donors. (C). All data are self-reported by donors.

Data were also evaluated from a total of 75 donors who supported the Amplify Hope campaigns. After contributing to a campaign, donors were asked to complete a survey (Multimedia Appendix 6) about themselves and the factors that influenced them to donate. A total of 64 out of the 75 (85%) donors were females compared with males (11 out of the 75, 13%), and the majority had some college and graduate degree (Figure 3). Moreover, 30 out of the 75 (40%) donors said they were friends of the individual raising money. Family or relatives represented the next largest segment at 26 out of 75 (35%), and 9 out of the 75 (12%) donors reported that they did not know the individual raising money.

A total of 46 out of the 75 (61%) donors heard about the crowdfunding campaigns through social media with Facebook as the most prominent platform in 67 out of 75 (89%) responses for learning about the campaign. However, there was a dramatic increase in the contribution amount when the medium used was word-of-mouth, email, and phone. This may be due to an increased personal connection through delivery of the message. To assess the effects of social pressure on charitable giving, respondents were asked whether they prefer to donate at the beginning of a campaign to impact the initial momentum in a campaign or at the end to see a campaign reach its goal. Respondents were asked to note their agreement with a series of statements. The question, “How important is it for you to fund crowdfunding projects that have already received substantial donations from others?” relates to the crowd effect when respondents view other’s donations and make a decision to fund a campaign that has received money from others. Most respondents (58 out of 75, 77%) thought it was at least moderately important, indicating that social recognition is a factor and the performance of the campaigns in general is key to why someone donates. Furthermore, respondents were asked, “How important is it to fund projects that are close to meeting their fundraising goal?” In total, 64 out of 75 (85%) reported moderately to very important. The amount raised appears to have an impact on the willingness of donors to get involved in funding a campaign.

### Education Through Crowdfunding

**Figure 4:**
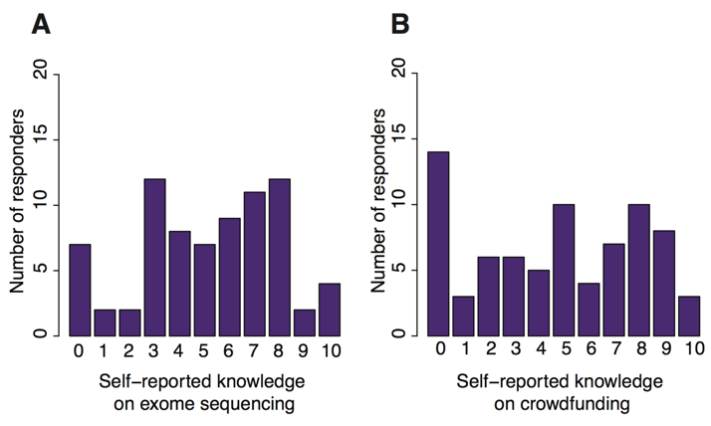
(A) Number of participants who reported each score for knowledge on exome sequencing. (B) Number of participants who reported each score for knowledge on crowdfunding.

The 86 study applicants were asked to report the knowledge on exome sequencing and crowdfunding on a scale of 0 to 10, with 0 referring to no knowledge. Respondents were more familiar with sequencing than crowdfunding. Among 86 applicants, 13 reported they had no knowledge of crowdfunding compared with 6 who were unfamiliar with sequencing (see Figure 4). When the selected 10 participants were asked, “Participating in this crowdfunding campaign has helped me to better understand the genetic test,” campaign organizers reported either agreeing or strongly agreeing.

Donors were asked to report their knowledge on genomics before and after donating to the campaigns. In total, 58 responses were collected. Majority of the donors self-reported they had limited knowledge of genomics before donating to the campaigns. When asked before the campaign to rate their knowledge of genomics on a scale from 0 (no knowledge) to 10 (expert), the average score was 2. After participating in the campaign, the average score increased by 84% with about 9 out of 58 (16%) scoring more than a 7. There were 24 (43% of total) respondents who scored “0” before the campaign, and this decreased by 71% to only 7 respondents afterward. The average new score for those who scored “0” before donating to the campaign was approximately 2.2.

Across all campaigns, all donors gained self-reported genomics knowledge by an average of 100% or more. To further examine the knowledge increase in genomics, respondents were asked if they understood what the funds of the project went toward and 51 out of 58 (87%) of the donors knew how the funds would be used, whereas 7 out of the 58 (7%) did not. When asked, “After donating to this campaign how interested are you in learning about genomics and personalized medicine,” 36 out of the 58 (62%) donors were interested in genomics and learning more about personalized medicine, whereas 7 out of the 58 (7%) were not interested. Lastly, donors were asked about their attitudes on the most effective ways to learn about genetic sequencing and genomics *.* Across all age groups, 46 of the 75 (61%) respondents felt videos were the most effective learning tool, followed by fact sheets/reports/technical papers and case studies.

## Discussion

### Limitations

Although the study showed several potential benefits of a crowdfunding approach to raise money for WES, there are also several limitations. One challenge inherent with rare or undiagnosed diseases is the small patient population. In addition, each participant in the Amplify Hope study had to have a genetic etiology consistent with rare undiagnosed diseases as confirmed by physician support for WES.

Other limitations of using a crowdfunding approach are the time and effort involved to reach individuals in a social network and to ask to support the campaign or share the campaign with their contacts. If a campaign organizer has a limited network of contacts, this could negatively affect the outcome. Another possible limitation is that individuals that are crowdfunding for WES may be the patient themselves and have medical limitations that prevent them from fully engaging in the crowdfunding process to achieve a successful outcome. A larger study using different milestones and improved methodologies would be needed to validate these conclusions with statistical power. With this, future researchers can continue to study this method or preference of instruction to provide information to educate the public on genomics and personalized medicine. Additional research will be needed, however, to assess the effectiveness of other instruction methods that were not included in this study.

### Principal Findings

The campaigns that achieved complete funding during the 30-day online crowdfunding campaign study period shared several characteristics. Individual campaign organizers who reached their campaign goals engaged more during the training program before the start of the crowdfunding campaign, as compared with those that did not achieve their campaign goals. These organizers read our shared Amplify Hope educational materials, followed recommended guidelines, and perhaps most importantly initiated communication via phone calls and emails to their networks before the campaign launch. In other words, the successful campaigners frontloaded their campaigns. They also actively engaged their network and donors by providing updates throughout the campaigns rather than trailing off.

Social media was important in connecting donors to the crowdfunding campaigns. In particular, Facebook was reported as the way many donors learned about the crowdfunding campaigns. Friends and family were the top sources of donations to the campaigns, supporting research that a strong network of individuals and larger social network equates to a higher crowdfunding success rate. Mollick and Kuppuswamy [10] in other studies examining crowdfunding for Kickstarter ventures found that having a large number of friends on online social networks is correlated with success. Demographic data obtained from our surveys revealed that women are more likely to donate to this research. This is consistent with the literature, which shows that a higher proportion of crowdfunding campaign donors are female. Greenberg and Mollick [11] and Marom et al [12] also found that women were considerably more likely than men to successfully raise capital for business ventures on Kickstarter, and argue that this is primarily due to the tendency of female donors to help other women. Additionally, we found that this donor segment was highly educated, with 66 out of 86 (76%) reporting having a bachelor’s degree or higher.

We also sought to determine the factors that influenced donors to support the crowdfunding campaigns for WES. We found that if a campaign is close to a deadline and has not reached their goal, donors are more likely to give money to help them reach their goal before the deadline. This is consistent with Kuppuswamy and Bayus [13], who found that in terms of monetary targets, donors on Kickstarter are more likely to support projects that are near their goals, viewing these projects as more likely to be successful, and that nearly all projects on Kickstarter that reach 50% of their fundraising goal are eventually fully funded. They also find that successful projects on Kickstarter are likely to have a public or private update near their campaign’s target end date.

Through online campaign summaries and links to educational resources, crowdfunding campaigns may increase knowledge regarding genetic sequencing, particularly as it relates to undiagnosed and rare diseases. As most of our participants and donors rely on social and electronic media, we hypothesized that the crowdfunding campaign and social media use leads to an increase of knowledge. We used postcampaign surveys to determine if the crowdfunding campaigns increased participants’ and donors’ knowledge of genomics or increased interest in learning about it. It is likely that participants had better sequencing knowledge due to the prerequisite of the study of physician-recommended WES, given this parameter, the patients had probably discussed the need for this testing with their physician. Majority of the donors had limited knowledge of genomics before donating, and across all campaigns, donors gained self-reported genomics knowledge by an average of 100% or more. The campaigns appeared to spur an interest in learning more about personalized medicine—46 out of the 75 (61%) donors were interested in learning more about genomics and personalized medicine. Postcampaign surveys were used to ask organizers about education on genetic sequencing as a result of having organized and executed a campaign. When asked, “Participating in this crowdfunding campaign has helped me to better understand the genetic test,” campaign organizers reported either agreeing or strongly agreeing. Our findings are consistent with Facio et al [14], in that there is interest in learning about genome sequencing and a perceived value in that knowledge. In addition to reporting a desire to learn more about genomics, donors in Amplify Hope also stated a preference as to how they would like to learn. When asked what method(s) would be most effective to learn more about genomics—video, webinars, fact sheets/technical papers, e-newsletters, or case studies—46 of the 75 (65%) respondents reported videos as their first-choice learning format. This supports the notion that videos are effective at communicating messages in crowdfunding campaigns and are the preferred method for continuing education on genetic sequencing and genomics among respondents.

Although crowdfunding has become a more popular means of donating in recent years, there is still a stigma associated with asking friends and family for donations for personal medical causes. We found that to be successful, the campaign organizer should actively participate in the process of outreach and have a higher degree of comfort with social networking as reported by the study participants.

### Future Works

This study showed that crowdfunding campaigns have the potential to benefit individuals with rare diseases seeking funding for various diagnostic and treatment options. The study highlights a self-reported donor population that is willing to fund research for friends, family, or strangers afflicted with a rare disease. Additionally, both donors and participants expressed a desire to not only learn more about personalized medicine and genomics, but there was additional specificity and preference on how to learn. The method of instruction through video is preferred by all respondents of the surveys. With this, future researchers can continue to study this method or preference of instruction to provide information to educate the public on genomics and personalized medicine. Additional research will be needed, however, to assess the effectiveness of other instruction methods that were not included in this study.

Crowdfunding offers a different approach to fundraising. The biggest difference between crowdfunding and traditional fundraising is that there are many smaller monetary donations in crowdfunding. Crowdfunding for scientific initiatives such as raising money for WES allows donors to choose the cause or person they would want to contribute to and they can view updates and continue to donate multiple times. There can be a sense of connectivity to the research by viewing updates on the campaign.

## Appendix

Multimedia Appendix 1 Interest Form.

Multimedia Appendix 2 Getting started worksheet.

Multimedia Appendix 3 Consent form.

Multimedia Appendix 4 Doctor's note.

Multimedia Appendix 5 Crowdfunding best practices: steps & stories to help you launch a successful campaign.

Multimedia Appendix 6 Donor's survey.

## Abbreviations

RG: Rare Genomics
WES: whole exome genetic sequencing
