# Metadata Correction: Engaging a Community for Rare Genetic Disease: Best Practices and Education From Individual Crowdfunding Campaigns

**DOI:** 10.2196/10707

**Published:** 2018-04-27

**Authors:** Romina Alicia Ortiz, Steven Witte, Arvin Gouw, Ana Sanfilippo, Richard Tsai, Danielle Fumagalli, Christine Yu, Karla Lant, Nicole Lipitz, Jennifer Shepphird, Fidelia B Alvina, Jimmy Cheng-Ho Lin

**Affiliations:** ^1^ Rare Genomics Institute Downey, CA United States

In the paper by Ortiz et al, “Engaging a Community for Rare Genetic Disease: Best Practices and Education From Individual Crowdfunding Campaigns” (Interact J Med Res 2018;7(1):e3), author Nicole Lipitz’s last name was incorrectly spelled as “Lipintz”. This has now been corrected.

The corrected article will appear in the online version of the paper on the JMIR website on April 27, 2018, together with the publication of this correction notice. Because this was made after submission to PubMed, PubMed Central, and other full-text repositories, the corrected article also has been re-submitted to those repositories.

