# Supplementary material for: Engaging a Community for Rare Genetic Disease: Best Practices and Education From Individual Crowdfunding Campaigns
Source: Interact J Med Res. 2018 Feb 5;7(1):e3. doi: 10.2196/ijmr.7176 (PMC5818677; doi:10.2196/ijmr.7176)
Supplement: Multimedia Appendix 1 [file ijmr_v7i1e3_app1.pdf]

Amplify Hope Initiative

* 1. Name

* 2. Email

3. Phone Number

* 4. Who is this sequencing for?

- Self
- Child
- Spouse
- Friend
- Please enter the name of the patient

* 5. Do you understand what exome sequencing is? 10 being expert

0	1	2	3	4	5	6	7	8	9	10

* 6. Do you know what crowdfunding is? 10 being expert

0	1	2	3	4	5	6	7	8	9	10

* 7. Do you have a physician who you are working with who can order the test?

- No
- Yes
- If yes, please provide their name and email
