# Supplementary material for: Engaging a Community for Rare Genetic Disease: Best Practices and Education From Individual Crowdfunding Campaigns
Source: Interact J Med Res. 2018 Feb 5;7(1):e3. doi: 10.2196/ijmr.7176 (PMC5818677; doi:10.2196/ijmr.7176)
Supplement: Multimedia Appendix 2 [file ijmr_v7i1e3_app2.pdf]

Getting Started Worksheet

* 1. Participant Email Address

2. Name of Patient

3. Date of Birth of Patient

	MM		DD		YYYY
Date		/		/	

4. Name of Applicant

5. Relationship of Applicant to Patient

- Self
- Mother
- Father
- Aunt or uncle
- Grandparent
- Other (please specify)

6. Participant Address

Address [ ]

Address 2 [ ]

City/Town [ ]

State/Province -- select state --

ZIP/Postal Code [ ]

Country [ ]

7. Clinical Referral (if applicable)

- Physician
- Geneticist
- Genetic Counselor

Provide their contact information and name of clinic/hospital

[ ]

8. Laboratory Referral

- Baylor Miraca Genetics Laboratories
- Ambry Genetics Laboratory
- Other (please specify)

[ ]

9. What is your gender?

- Male
- Female
- Other
- Prefer not to dosclose

10. What is your age?

- ◯ 17 or younger
- ◯ 18-20
- ◯ 21-29
- ◯ 30-39
- ◯ 40-49
- ◯ 50-59
- ◯ 60 or older

11. What is the highest level of school you have completed or the highest degree you have received?

- ◯ Less than high school degree
- ◯ High school degree or equivalent (e.g., GED)
- ◯ Some college but no degree
- ◯ Associate degree
- ◯ Bachelor degree
- ◯ Graduate degree

12. Which of the following categories best describes your employment status?

- ◯ Employed, working 40 or more hours per week
- ◯ Employed, working 1-39 hours per week
- ◯ Not employed, looking for work
- ◯ Not employed, NOT looking for work
- ◯ Retired
- ◯ Disabled, not able to work

13. What social media platforms do you have accounts on?

- [ ] Facebook
- [ ] Twitter
- [ ] Instagram
- [ ] LinkedIN
- [ ] Google+
- [ ] Tumblr
- [ ] Flickr
- [ ] Pinterest
- [ ] Path
- [ ] Vine
- [ ] Youtube
- [ ] Redditt

14. How often do you check social media?

- ○ More than 20 times a day
- ○ Between 10 to 20 times a day
- ○ Less than 10 times a day
- ○ A few times a week
- ○ A few times a month or even less
- ○ I don't use social media

15. How many friends do you have on Facebook?

- None
- 1-50
- 51-100
- 101-250
- 251-500
- 501-1000
- 1000-2500
- more than 2500

16. How many followers do you have on Twitter?

- None
- 1-50
- 51-100
- 101-250
- 251-500
- 501-1000
- 1000-2500
- more than 2500

17. How often do you post on social media per week?

- More than 20 times a day
- Between 10 to 20 times a day
- A few times a week
- A few times a month or even less
- I don't use social media

18. How do you share normal updates about your rare disease journey? (Check all that apply)

- [ ] Facebook
- [ ] Twitter
- [ ] Instagram
- [ ] LinkedIn
- [ ] Google+
- [ ] Tumblr
- [ ] Flickr
- [ ] Pinterest
- [ ] Path
- [ ] Vine
- [ ] Youtube
- [ ] Redditt

19. Are you part of a community or group? (Check all that apply)

- [ ] Religious/faith
- [ ] Cultural
- [ ] Career Association
- [ ] Volunteer
- [ ] Other (please specify)

20. How many times have you created a crowdfunding campaign to raise funds?

- ○ None
- ○ Once or Twice
- ○ Three to five times
- ○ More than 5

21. How many times have you donated to a crowdfunding campaign?

- None
- Once or Twice
- Three to five times
- More than 5

Getting Started Worksheet

22. Number of physicians patient has seen

23. Types of physicians patient has seen (Check all that apply)

- [ ] Clinical geneticist
- [ ] Gastroenterologist
- [ ] Cardiologist
- [ ] Neurologist
- [ ] Endocrinologist
- [ ] Pulmonologist
- [ ] Psychiatrist
- [ ] none
- [ ] Other (please specify)

24. Testing done for patient (Check all that apply)

- [ ] Comparative Genome Hybridization
- [ ] Karyotype / Chromosome analysis
- [ ] Gene Sequencing (e.g. single or panel)
- [ ] DNA Microarray
- [ ] MRI
- [ ] Whole Exome or Genome Sequencing
- [ ] none
- [ ] Other (please specify)

25. Types of support services used for patient (Check all that apply)

- [ ] Physical Therapy
- [ ] Speech & Language Therapy
- [ ] Behavioral Therapy
- [ ] Occupational Therapy
- [ ] Audiology
- [ ] none
- [ ] Other (please specify)

Getting Started Worksheet

In order to be accepted to this study, a signed consent form must be uploaded and submitted.

Please click here to take you to the consent form . To scroll down in the form, use your keyboard arrows.
