# Supplementary material for: Engaging a Community for Rare Genetic Disease: Best Practices and Education From Individual Crowdfunding Campaigns
Source: Interact J Med Res. 2018 Feb 5;7(1):e3. doi: 10.2196/ijmr.7176 (PMC5818677; doi:10.2196/ijmr.7176)
Supplement: Multimedia Appendix 3 [file ijmr_v7i1e3_app3.pdf]

Rare Genomics Institute

INFORMED CONSENT TO PARTICIPATE IN A RESEARCH STUDY
Crowdfunding Participant

Participant's Name: ____________________________________
Project Title: Amplify Hope Initiative
Principal Investigator: Jimmy Lin, M.D, PhD.
Research Team Contact: Romina Ortiz, M.H.S, romina.ortiz@raregenomics.org

This form describes the purpose of this research study, what you will be asked to do during the study, and about your rights as a research participant. It helps you decide if you want to participate. By signing this form and uploading it to our study application you are agreeing to participate in this study.

- If you have any questions about anything in this form, you should ask the research team for more information.
- You may also wish to talk to your family or friends about your participation in this study.

Do not agree to participate in this study unless the research team has answered your questions and addressed your concerns to your satisfaction. Do not agree to participate unless you have weighed the risks and benefits and decide that you want to be part of this study.

WHAT IS THE PURPOSE OF THIS STUDY?

This is a research study. We invite you to participate in this research study because you are trying to raise funds to pay for genetic sequencing.

The purpose of this study is to help the participants and families raise funds for genetic sequencing and measure the impact of community engagement with crowdfunding. The work involves surveys to explore what factors contribute to successful crowdfunding, and whether it can stimulate increased engagement with scientific questions. Additionally, we will explore innovative strategies to promote engagement through crowdfunding: through developing educational and communication material, comparing different platforms, and leveraging social networks and incentive engineering. Outputs from the project will include online education and training content, academic publications, and culminate in a white paper to outline the potential of crowdfunding to emerge as a major source of funds for scientific research and present a roadmap for crowdfunding platforms and researchers to take advantage of this emerging mechanism.

WHAT WILL HAPPEN DURING THIS STUDY?

Your current sequencing site has informed you of this opportunity because they have identified you as eligible for this study. You are eligible if you have a physician that will prescribe genetic sequencing for the patient, and you would like to raise funds to pay for the test.

If eligible and willing to participate, you shall:

1. Apply to this study through our online application form
2. Sign and upload this consent form to your application
3. Participate in our informational Webinar where we will discuss the study and steps to prepare for crowdfunding
4. Be randomly assigned to a crowdfunding platform matched to its most effective strategy
5. Create your crowdfunding campaign on the designated crowdfunding platform
6. Utilize only the assigned crowdfunding strategy to raise the funds during the 30 days of fundraising
7. Understand that after 30 days of fundraising, the amount raised will be transferred from Rare Genomics Institute to your genetic sequencing site
8. Understand that after fundraising is complete, data of the characteristics of your crowdfunding campaign will be collected and analyzed
    a. Such data includes:
        i. **Demographic data**: collected from your study application. The patient's and participant's name, your relationship to patient, the patient's date of birth, your email, your home address, the laboratory you were referred from, social media activity, previous crowdfunding experience and patient diagnostic journey.
        ii. **Campaign data**: preparation for raising funds, time to complete fundraising, number of social connections contacted by donors, rate of contacting social connections, number of donors, average donation amount, percent of users that follow internet links to online educational information.

<u>Will you save my demographic information or crowdfunding research data to use in future research studies?</u>

We will only ask for basic demographic information in our application and collect data on crowdfunding metrics as described above. The crowdfunding metrics will be pre-determined by the designated crowdfunding platform.

You will only be identified/your information will only be accessed through a number assigned to your case, which will not include any of your personal health information or identifiers.

Please place your initials in the blank next to Yes or No for each of the questions below:

My application data may be stored and used for the study as described above.

_____ Yes **_____ No**
Initials **Initials**

My crowdfunding data may be shared with other investigators and used by these investigators for the future research as described above.

_____ **Yes** _____ **No**
Initials **Initials**

HOW MANY PEOPLE WILL PARTICIPATE?
Approximately 50 people will take part in this study conducted by investigators at Rare Genomics Institute

HOW LONG WILL I BE IN THIS STUDY?
Participants will be brought into the study starting in March 2015. You will receive coaching to create your crowdfunding sites and all campaigns will launch June 2015. The campaigns will be for 30 days and end July 2015. Data will be collected and analyzed until October 2015.

WHAT ARE THE RISKS OF THIS STUDY?
You may experience one or more of the risks indicated below from being in this study. In addition to these, there may be other unknown risks, or risks that we did not anticipate, associated with being in this study.

Likely / Common risks
Life Threatening: None

Serious: None

Mild: None

Less Likely / Less Common risks
Life Threatening: None

Serious: Emotional or psychological distress due to asking for donations

Mild: None

WHAT ARE THE BENEFITS OF THIS STUDY?
By participating, you will be coached to fully utilize a crowdfunding strategy that leading experts in the field have identified as successful. This will allow you to have support in reaching out to your network and ultimately raise funds for genetic test that you may otherwise not be able to afford.
Crowdfunding has had a transformative impact on charitable giving and entrepreneurial finance but has yet to translate this impact to scientific research. This project has the potential to stimulate crowdfunding in this sector and build a lasting impact of greater public involvement and engagement in the scientific enterprise, and a greater consideration of the big questions that follow.

WILL IT COST ME ANYTHING TO BE IN THIS STUDY?
Rare Genomics Institute does not charge money for its services. The amount of money

necessary will be pre-determined in conjunction with the crowdfunding and clinical sequencing sites and will include crowdfunding platform operational costs. Any money raised above the necessary amount determined for genetic sequencing will go to other families/individuals crowd funding with Rare Genomics Institute to pay for their genetic sequencing. If the necessary funds are not raised over the course of the initial study, an additional 60 days will be allowed to raise the money. If after the additional 60 days the necessary funds are still not raised, the amount that was raised will be transferred to the sequencing site to determine how to proceed on a case by case basis. Agreement for sequencing will be conducted between participants and their sequencing laboratory indicated in the completed application. Rare Genomics Institute is only helping participants raise funds and is not responsible or liable for paying any remaining funds necessary for sequencing.

WILL I BE PAID FOR PARTICIPATING?
You will not be paid for being in this research study.

HOW WILL YOU KEEP MY INFORMATION CONFIDENTIAL?
Participation in this research study will be public, as you will be using a crowdfunding website to raise funds publicly.

To help protect your confidentiality, all research data we will collect to analyze (demographic and crowdfunding campaign) will be assigned a study number that contains no identifying personal information. The data will be password protected and access will be restricted to Dr. Lin's Study Team members. If we write a report or article about this study or share the study dataset with others, we will do so in such a way that you cannot be directly identified. Once information is de-identified, it may be used and shared for other purposes not discussed in this consent form.

IS BEING IN THIS STUDY VOLUNTARY?
Taking part in this research study is completely voluntary. You may choose not to take part at all. If you decide to be in this study, you may stop participating at any time. However, any money raised will go to helping families crowdfunding with Rare Genomics Institute to pay for genetic sequencing. If you decide not to be in this study, or if you stop participating at any time, you will not be penalized or lose any benefits for which you otherwise qualify.

What if I decide to withdraw from the study?
You may withdraw by telling the study team you are no longer interested in participating in the study or you may send in a withdrawal letter.

If you decide to leave the study early, we will retain your demographic and crowdfunding data available but erase all your personal identifying information from our database.

Will I receive new information about the study while participating?
If we obtain any new information during this study that might affect your willingness to continue participating in the study, we will promptly provide you with that information.

Can someone else end my participation in this study?
Under certain circumstances, the researchers might decide to end your participation in this research study earlier than planned. This might happen because you do not attempt to utilize the crowdfunding strategy and platform randomly assigned to you.

WHAT IF I HAVE QUESTIONS?
We encourage you to ask questions. If you have any questions about the research study itself, please contact: Romina Ortiz, M.H.S (romina.ortiz@raregenomics.org).

If you sign this form:

- You agree that you will participate in the study and abide by the crowdfunding strategy and platform assigned to you.
- You authorize the use of your demographic and crowdfunding information for this research.
- Your signature and this form will not expire as long as you wish to participate.
- You may later change your mind and withdraw from the study, however the data already collected will be retained and analyzed by Rare Genomics Institute.
  - To revoke your authorization, contact Romina Ortiz at (romina.ortiz@raregenomics.org).
    - **If you revoke your authorization:**
      - The research team may only use and share information already collected for the study.
      - Your information may still be used and shared if necessary for safety reasons.
      - You will not be allowed to continue to participate in the study.

This consent form is not a contract. It is a written explanation of what will happen during the study if you decide to participate. You are not waiving any legal rights by agreeing to participate in this study. Your signature indicates that this research study has been clearly explained to you, that your questions have been answered to your satisfaction, and that you agree to take part in this study. You will receive a signed copy of this form.

______________________________________ ____________
(Signature of Participant) (Date)

______________________________________ ____________
(Participant's name – printed) (Date)
