# Supplementary material for: Engaging a Community for Rare Genetic Disease: Best Practices and Education From Individual Crowdfunding Campaigns
Source: Interact J Med Res. 2018 Feb 5;7(1):e3. doi: 10.2196/ijmr.7176 (PMC5818677; doi:10.2196/ijmr.7176)
Supplement: Multimedia Appendix 4 [file ijmr_v7i1e3_app4.pdf]

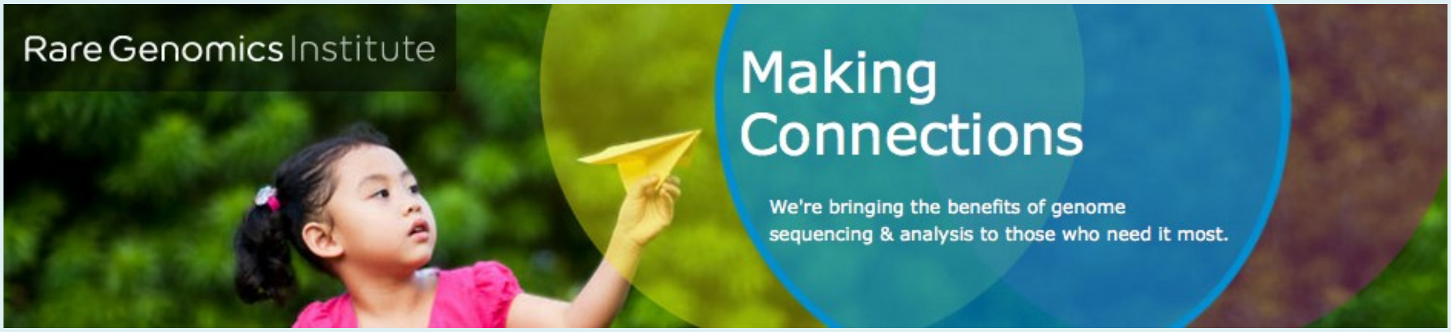

PHYSICIAN NOTE FOR SEQUENCING

PHYSICIAN INFORMATION

Name:

Email

Street address

City

State

INFORMATION TO BE RELEASED TO

Organization: Rare Genomics Institute

Address: 2657 Annapolis Road, Suite G #105, Hanover, MD 21076

Contact Name: Romina Ortiz

Contact Email: romina.ortiz@raregenomics.org

This patient has enrolled in our new Amplify Hope Initiative, where they will crowd fund to raise the money necessary to receive whole exome sequencing from one of our two laboratory partners, Ambry Genetics or Baylor Miraca Genetics Laboratories. For more information, visit our website: http://amplifyhope.raregenomics.org/

I understand that the information I give is my own statement based on my knowledge of medical information pertaining the patient, and immediate family as well as my intent to help the child clinically.

I hereby recommend ____________ for genetic sequencing and will be in contact with Rare Genomics Institute, the sequencing laboratory and possible research scientists associated with the case to ensure the referral to sequencing, implementation of sequencing and clinical interrogation of results to determine the best way to help the child. I acknowledge I have fully reviewed and understand the contents of this authorization form.

Name

Signature

Date
