# Supplementary material for: Engaging a Community for Rare Genetic Disease: Best Practices and Education From Individual Crowdfunding Campaigns
Source: Interact J Med Res. 2018 Feb 5;7(1):e3. doi: 10.2196/ijmr.7176 (PMC5818677; doi:10.2196/ijmr.7176)
Supplement: Multimedia Appendix 5 [file ijmr_v7i1e3_app5.pdf]

CROWDFUNDING
BEST PRACTICES

STEPS & STORIES
TO HELP YOU
LAUNCH A
SUCCESSFUL
CAMPAIGN

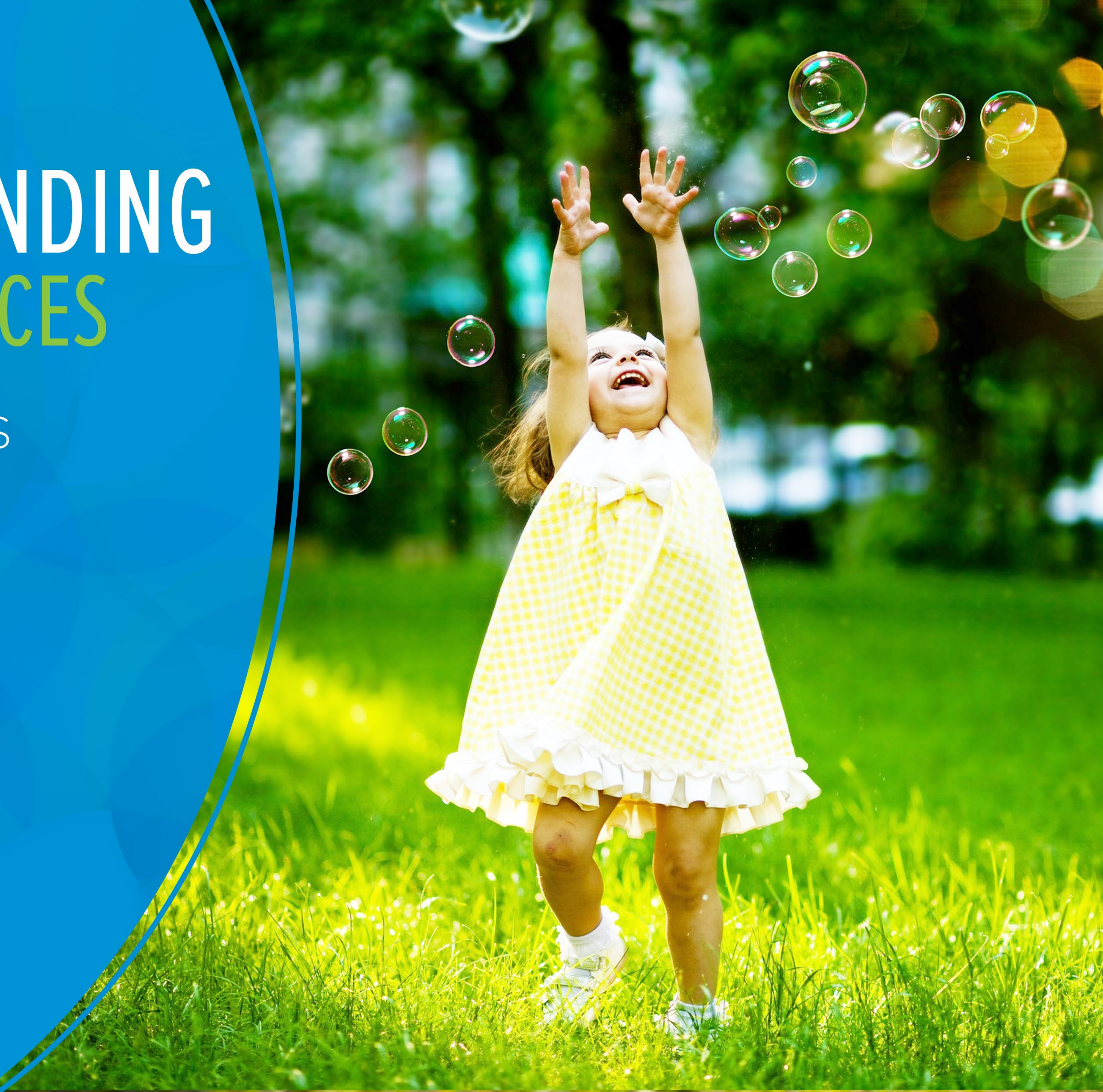

Rare
Genomics
Institute

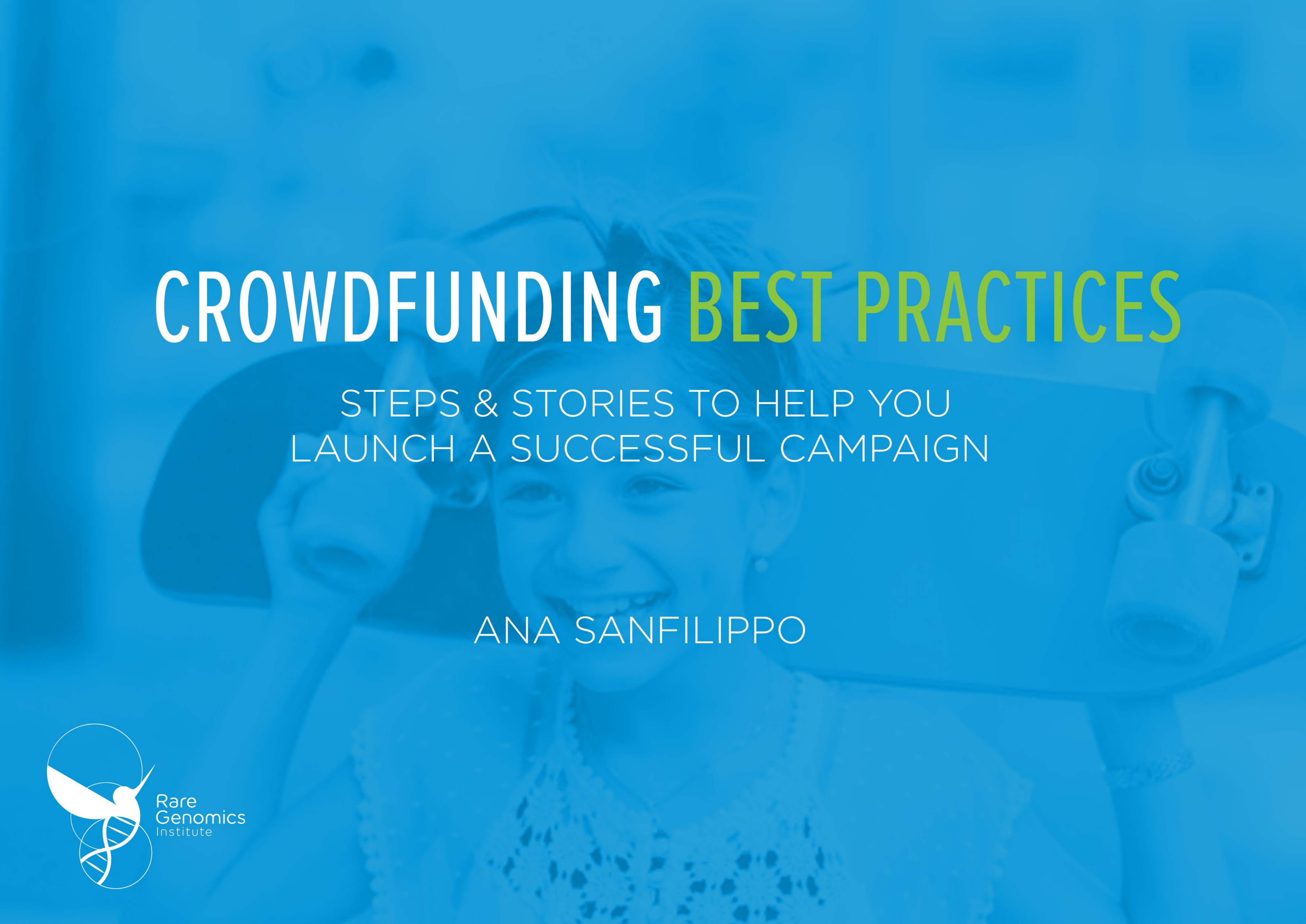

CROWDFUNDING BEST PRACTICES

STEPS & STORIES TO HELP YOU LAUNCH A SUCCESSFUL CAMPAIGN

ANA SANFILIPPO

Rare Genomics Institute

CONTENTS

5 Crowdfunding: The Big Picture

10 Pre-Game Plan

- What to do before the campaign begins?

14 Messaging is Key to the Campaign

- Cultivate a Community
- Telling Your Story

20 Team: Recruit Your Team and Lock in Beginning Donations

- Share Connections to Create Your Peripheral Team

27 Write an Awesome Crowdfunding Email

32 Phone Calls are Important

34 Goal Setting for Fundraising

38 How to Make a Great Crowdfunding Video

48 What's a Great Picture?

51 What About Perks and Gifts

54 Launch Day

- What to do the first 24 hours?

58 Running the Campaign

- Media
- Updates and Engagement
- Timeline
- Offline Fundraising Efforts
- Advice and Feedback

66 The Final Push to the Campaign Goal

69 Appendix

72 Contributors

Acknowledgements

Many people contributed to the development of this book. Crowdfunding experts shared their advice and experiences. In particular, I would like to thank Ethan Austin, Breanna DiGiammarino, Adam Griff, Annette Hayswirth, Elizabeth Iorns, Nick Karolidis, Denny Luan, Molly Lindquist, Andrea Lo, Luke Miner, Jamie McDonald, Sandip Sekhon, Nick Sireau, Devin Thorpe, and Rob Wu for sharing their expertise. I would also like to thank Sam De Brouwer, Zsuzsanna Darvai, Ignacia Garcia, Kimmie Ng, Glenn O'Neill, Susanne Shaw, and Jeneva Stone for sharing insights about their own crowdfunding campaigns.

Thank you to the Rare Genomics Team who offered comments, assisted in the editing, proofreading and design. In particular, Karla Lant, Mike Russo and Christine Yu.

On behalf of Rare Genomics Institute, we would like to thank the John Templeton Foundation for the grant that funded this work.

Crowdfunding: The Big Picture

Rare Genomics Institute

CROWDFUNDING: THE BIG PICTURE

What is crowdfunding? As the name suggests, crowdfunding involves asking a crowd of people to donate money to an individual project or campaign. Crowdfunding is another type of fundraising, but this fundraising is done online.

Crowdfunding works because a large number of people donate what are usually smaller amounts of money to a project or venture. This is the cause behind the campaign. Crowdfunding is used to raise money for a wide range of projects from businesses to films to personal medical expenses. There are different kinds of projects and campaigns, but we are going to focus on crowdfunding for medical campaigns.

Crowdfunding has created a paradigm shift in fundraising. Traditional fundraising involved larger dollar donations, usually from a few philanthropic individuals or investors. Crowdfunding allows for small donations from many individuals anywhere around the world.

Smaller amounts by many. The common ability of many to donate small amounts of money to a cause, coupled with the power of connectivity via the Internet means that anyone anywhere can support projects around the world. It also means that anyone can share the information about the campaign with their own network. Crowdfunding provides entrepreneurs, small business owners, and individuals an alternate funding mechanism at a time when the traditional sources of funding have become more difficult to obtain.

Social media. Crowdfunding is not a new concept, but over the last few years, it has become more widely used and publicized through the media. What usually starts out as a small circle of friends and family knowing about a fundraising cause can quickly spread to thousands

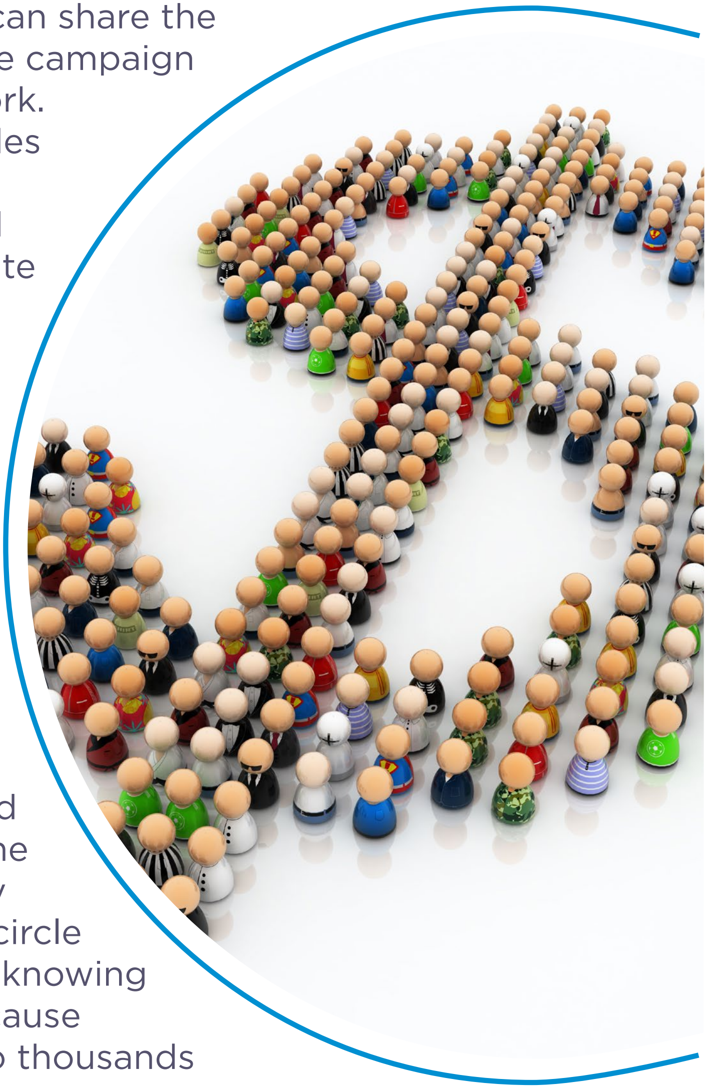

of people using the power of social media. The biggest benefit of a crowdfunding campaign is that a lot of money can be raised in a short amount of time.

There are many choices when it comes to crowdfunding platforms with more than 500 different sites as options. An important difference to consider is the two models, "**All or Nothing**" versus "**Keep It All.**" In the first model, you must reach the goal amount you set in order to keep the money raised. In the second model, you keep everything you raised, minus any fees, even if you do not reach your goal.

In this ebook, we will evaluate some of the most successful crowdfunding campaigns and share advice from the experts and the founders of the most popular crowdfunding platforms. Our focus is crowdfunding for personal medical causes and scientific research. Their insights are synthesized into this guide of best practices to show you how to run a successful campaign.

WHAT YOU WILL LEARN:

Crowdfunding: The Basics

Crowdfunding involves asking a crowd of people online to donate money to an individual project or campaign. It works because a large number of people donate what are usually smaller amounts of money to a project or venture. Crowdfunding is very effective for medical campaigns.

Before the Campaign Begins

- Set a clear, realistic, measurable goal and timeframe
- Engage in detailed planning 90 days before your start date
- Create your message, identify your target community, recruit your team, and figure out how to tell your story
- Strategize about how to create energy and group momentum to prompt more activity; experts agree that successful crowdfunding campaigns boil down to knowing the right problem, the right approach, the right audience, and timing all of these factors appropriately
- Arrange initial commitments from your circles with the goal of securing 30% funding on the start date
- Create your media: high-quality photos, a video, and written materials will be essential

During the Campaign

- Hold a launch party on Day One
- Provide regular, detailed updates
- Use social media for the campaign every day
- Engage in offline fundraising and link it to the online work
- Contact the media
- Elicit feedback and act on it
- Make a strong final push at the end of the campaign

After the Campaign

- Thank your donors and provide them with anything you promised to them
- Revise for next goal, as needed

Crowdfunding has prompted a culture change in the scientific community. The advantage of this technique is that it allows researchers the resources they need to do important work. Crowdfunding represents the democratization of research and a promising path towards general scientific engagement for members of the community.

Planning Crowdfunding Campaigns

Experts agree that successful campaigns boil down to knowing the right problem, the right approach, the right audience, and the right timing. If you mix these elements together in the correct way, you will be successful; this is why planning is essential.

When planning remember to ask and answer these questions:

- How will I present this case? Consider message, branding, and visual factors here.
- Why is this problem important? Explain why people should care about it and what effect funding will have.
- Why is this timing important? Explain why you need funding at this point: mention the need for testing, an upcoming procedure, or a roadblock to finding cures or medicine that exist.
- Why should people fund this project? Explain what will happen if they fund you, what will you be able to do, and what the broader impact may be.

Think about these critically to ensure that you make a connection with your audience.

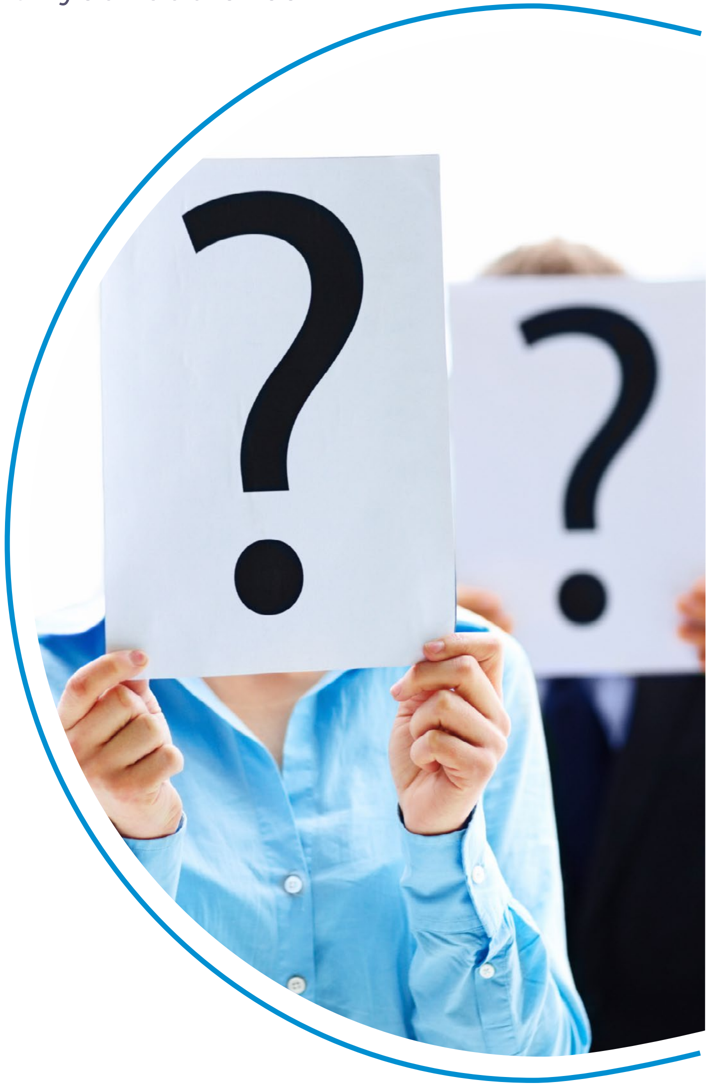

Pre-Game Plan

Rare Genomics Institute

PRE-GAME PLAN

What to do before the campaign begins?
Because crowdfunding is so different from other kinds of fundraising, the key to a successful campaign is **planning**. Focusing a cause online by crowdfunding can create a momentum that relies on the power of the group to encourage others to donate to a campaign or cause.

One of the best indicators of a successful campaign is when it raises a lot of money before the start of the campaign. When the crowd sees a campaign that quickly raised funds, especially on the launch day, there is an energy and momentum that occurs. Often the crowd follows and donates more to the campaign. This is thanks to the energy and momentum of the group which prompts more activity.

So, how will you create this momentum and raise funds before the campaign even begins? **The secret is to get commitments early from your inner circle of supporters.**

Experts agree that successful campaigns boil down to the right mix of variables: **knowing the right problem, the right approach, the right audience and timing.** If you mix these elements together in the correct way, you will be successful.

Crowdfunding uses the core technology of the Internet and existing communities of people. Crowdfunding is just a technology that enables reach. This means that the biggest determinant of a successful campaign is how you present your case. Before the campaign begins, answer these questions: Why is this problem important? Why is this timing important? Why should you fund this project? Think about these critically to ensure that you make a connection with your audience.

> **"The game is won or lost before you set foot on the field. There are two important aspects to the campaign: content and distribution."**
> **—Ethan Austin,**
> **co-founder GiveForward**

As GiveForward, co-founder Ethan Austin explains with crowdfunding, "The game is won or lost before you set foot on the field. The two most important aspects to any crowdfunding campaign are content and distribution." Content tells the story. You have to paint the picture of what you

are going through for your audience. Successful campaigns carefully plan how the cause should be presented. Distribution means how the campaign is marketed.

Sometimes people don't really know what to think the first time they hear about a campaign. However when they see people talking about a campaign and donating to it, they will do the same. What are the most important lessons we can take away from a successful campaign? Ethan Austin said, "Personalization, promotion and persistence are the keys."

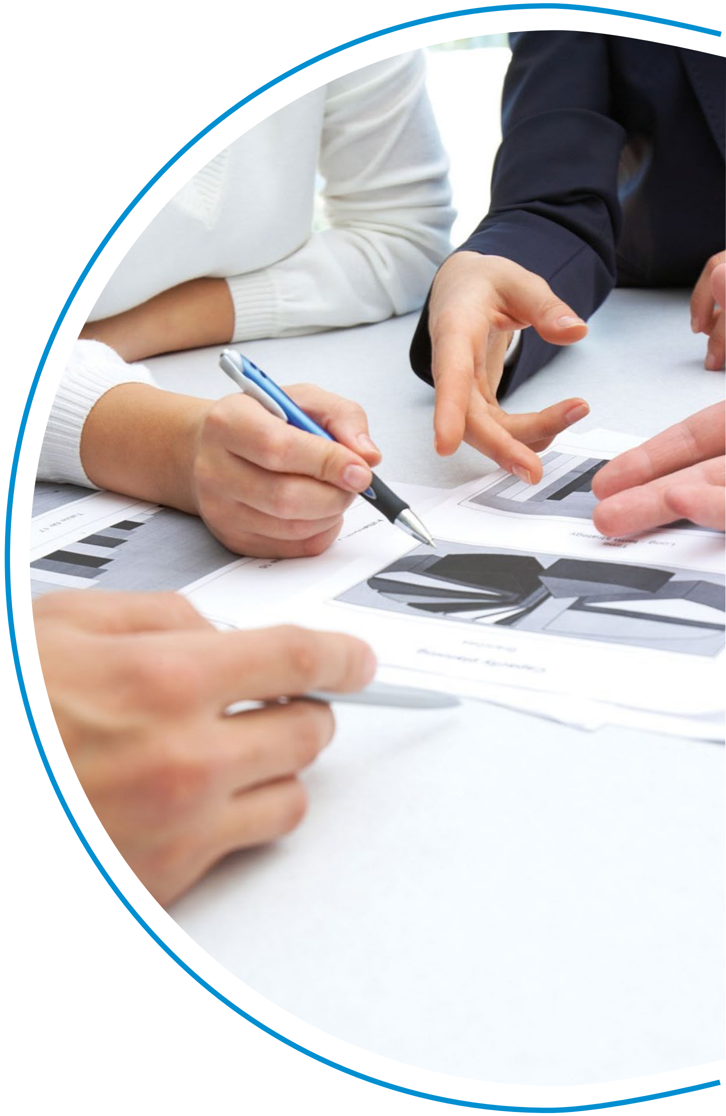

Case Study: Successful Planning

One example of a very successful Kickstarter campaign was the Coolest Cooler that raised $13.3 million in 52 days. They had 2 campaigns. In the first launch, only $100,000 was raised. Although this is a significant amount, it was not the goal they had hoped to reach.

In their second campaign, what changed that made them so successful? The content was basically the same for the cooler, so that did not change. However, they built up $100,000 dollars of commitment on Kickstarter in the first campaign for the cooler. They assembled a crowd of people that knew about it and supported it. When they launched it a second time, they had about 1000 fans of the product, so they seeded it. The first time they were outsiders, the second time they had a tribe of followers.

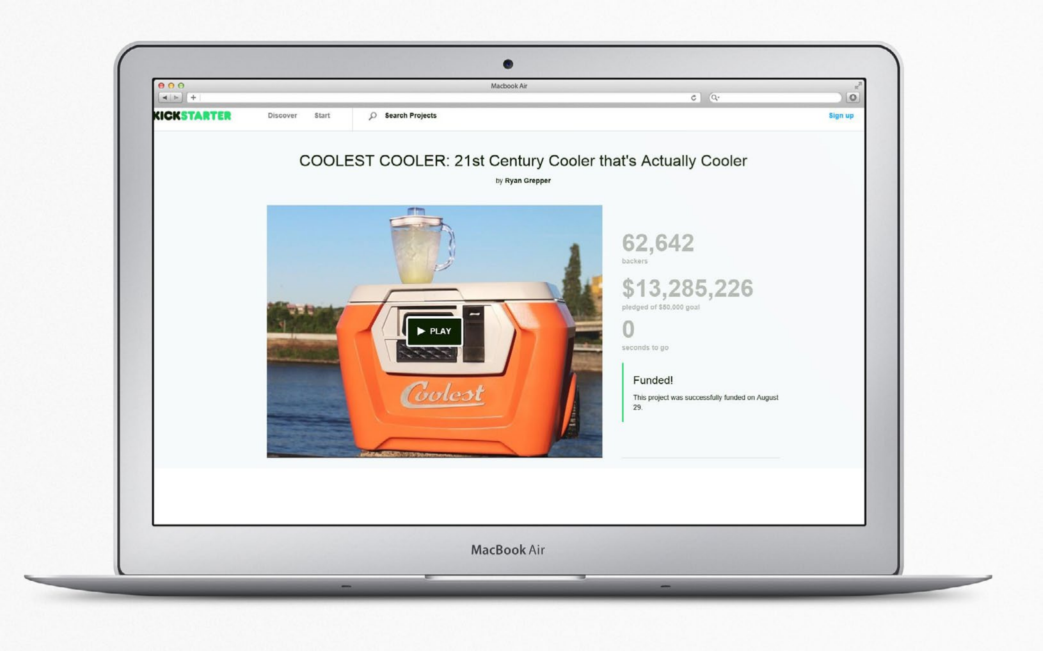

Messaging is Key to the Campaign

Rare
Genomics
Institute

Create your message and identify your target community

Create your message and identify your target community

During the Campaign

- Tailor your message for momentum. Strategize about how to create energy and group momentum to prompt more activity; experts agree that successful crowdfunding campaigns boil down to knowing the right problem, the right approach, the right audience, and timing all of these factors appropriately.
- Research the community. Find out which groups of people are most likely to support you.

Only well thought out, planned, and emotionally moving messages and campaigns take off, and your target audience heavily influences what message will be effective.

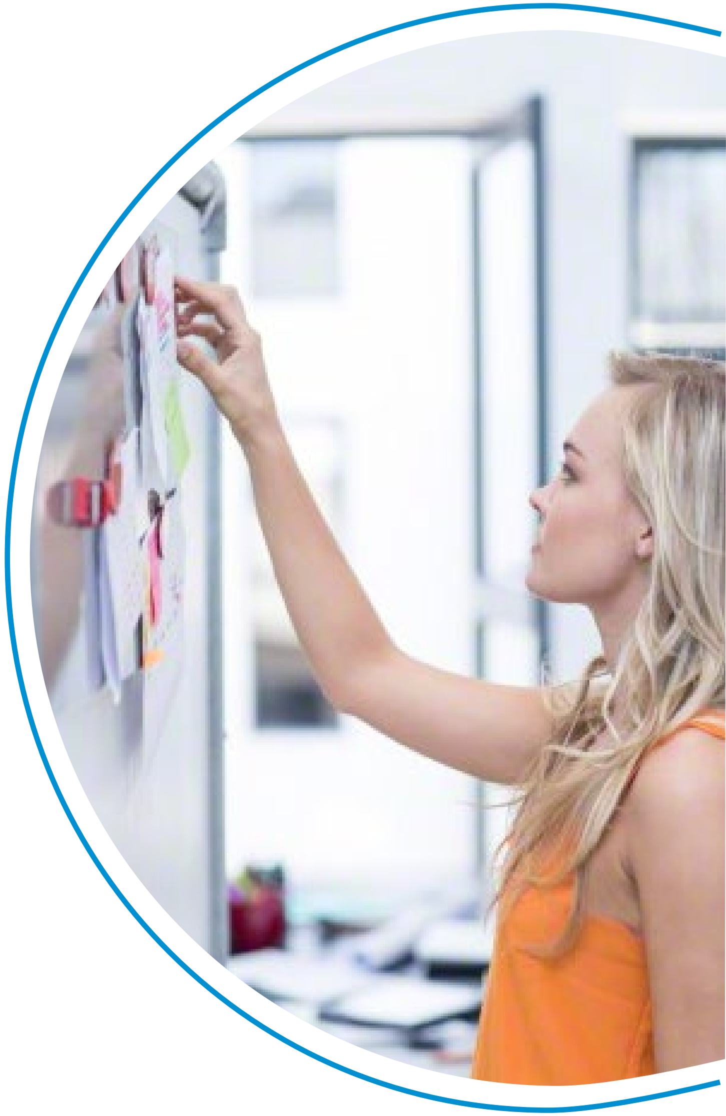

MESSAGING IS KEY TO THE CAMPAIGN

Messaging is the most important part of your campaign. Crowdfunding expert Devin Thorpe recommends that people with health problems plan their campaigns starting about 90 days before the campaign launch date. First, make sure crowdfunding will work for you.

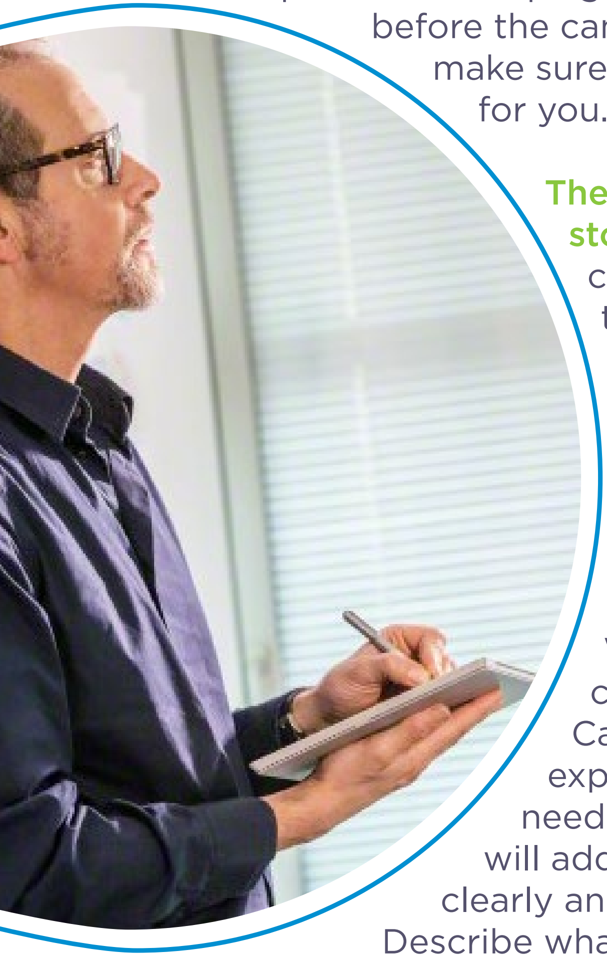

There has to be a strong story. It is important to craft the story so that the audience can readily understand the problem and know why there is a great need for them to support the campaign.

Simply explain the problem. Rob Wu, founder of the crowdfunding site CauseVox recommends explaining the problem or need that the money raised will address in simple terms. Do it clearly and in one or two sentences. Describe what the campaign is about, and tell potential donors how their efforts will make an impact.

Be transparent. It's important to be transparent and believable. People respond to emotion, so the campaign should state the need earnestly (so potential donors believe in it) and visually (so they can better understand how difficult the issue is). Finally, tell them how they can help. Give them a call to action and let them know when the campaign starts and ends.

Understand your target audience. Think about what influences them and speak their language in your messaging. Tailor the message to highlight the benefits supporting the campaign will have to that particular audience. With medical campaigns in particular, raising money for just one clinical trial may lead to major developments in another disease state or illness. Remind readers of this key benefit.

MESSAGING CHECKLIST:

- ☑ Find organizations and groups that have a connection to the cause
- ☑ Personalize the message
- ☑ Messaging should answer these questions:
  - ☑ Who am I?
  - ☑ Why am I raising money?
  - ☑ Why do I need help?
  - ☑ How will the money be spent?

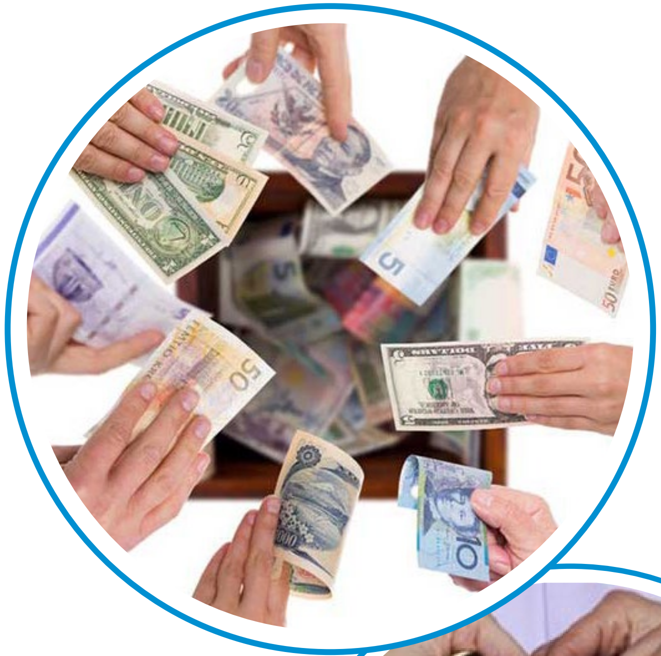

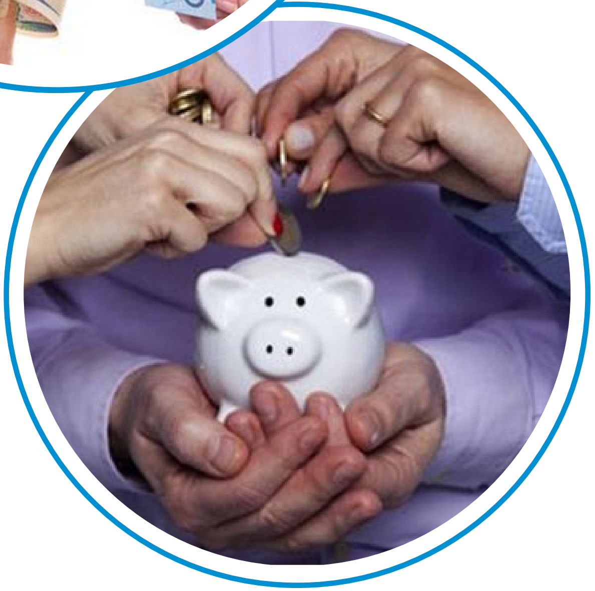

Cultivate a Community

Find groups of people that would want to help you. They could be associations, organizations involved in the illness or disease state, or church groups. Find groups of people that have a connection to the cause.

Remember to ask. It's important to clearly ask for what you want, and to make the amount relatable. As an example, state that $50 pays for one treatment. Personalize it so that the donor knows exactly where their $50 (or other amount) of money is going. Make the narrative describing the campaign and the person in need very clear.

For example, explain the illness that "Emily" has and what her experience has been like so far. Then encourage them to donate, reminding them specifically how that amount will help Emily.

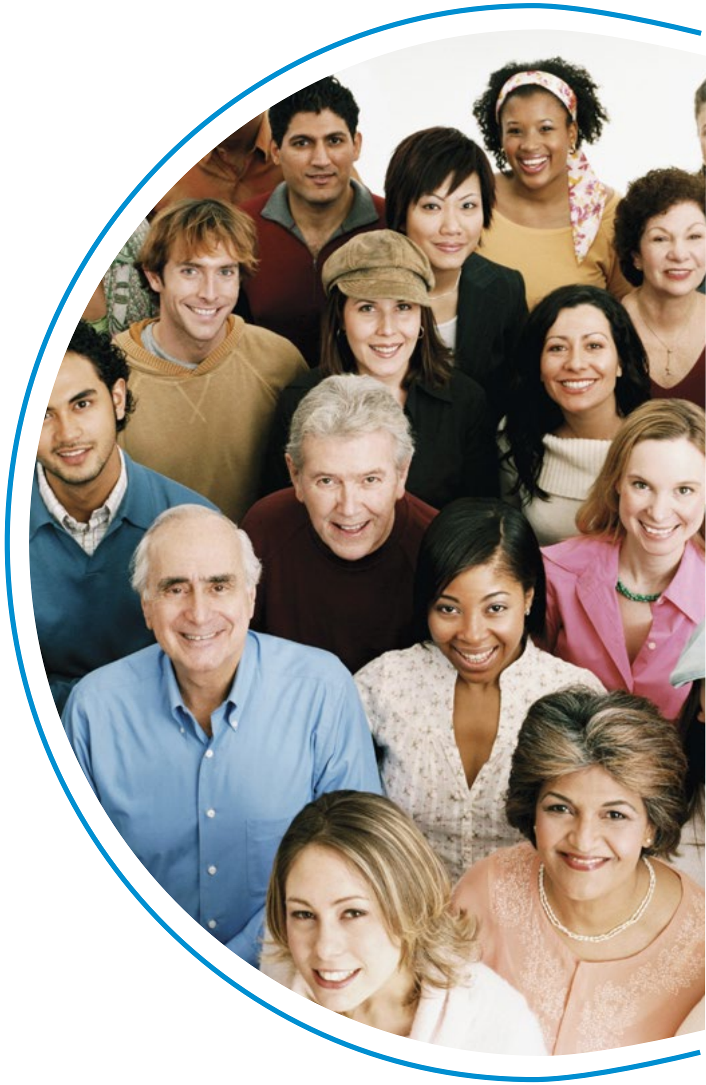

Telling Your Story

Understand that people have a short attention span and create your message with that in mind. When telling your campaign story, make sure you answer these questions: Who am I? Why am I raising money? Why do I need help? How will the money be spent? Take a step back and consider what would drive you to part with your money for someone else's campaign page. Then craft your message.

People can only digest small amounts of information. Do not use a huge block of text. Separate it into smaller sections that are easy to read and understand. Use headings.

Make people feel empathy. If a family is trying to raise money for a sick child who has a rare disease, explain how this illness affects their normal, everyday activities. Tell them what the life of this child is like. You want your audience to walk in your shoes and think, "Imagine if that hit me."

Keep these reasons at the forefront when you plan the messaging of your campaign. If your story is a good candidate for crowdfunding, next look at platforms. In the first 30 days of your planning period, think about all aspects of the campaign including media outreach. Gather high quality photos. The remaining 60 days leading up to the campaign launch, prepare and gather a team. If you have extended family and friends that are willing to work with you and are reliable, put a big team together.

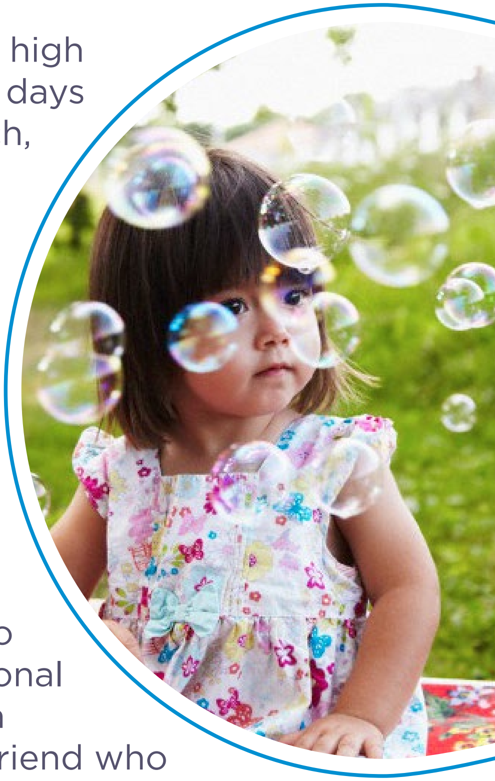

Why do people donate? When building your campaign, think about the many reasons that might prompt others to donate to it. Here are some reasons to consider. Maybe they can relate to the situation from their own personal experiences. Perhaps, they have a connection, a family member or friend who has a similar issue. If they know someone with the same problem, they can feel more connected to your cause and more likely to support it. They may just want to make a positive difference in someone else's life by donating. Or they may want to donate so they can be recognized for their donation publicly on the page or receive a thank you note or gift.

Team: Recruit Your Team and Lock in Beginning Donations

Rare Genomics Institute

TEAM: RECRUIT YOUR TEAM AND LOCK IN BEGINNING DONATIONS

- **Create your "A" team.** Identify your inner circle of family and friends and choose people who are reliable. Ensure you have complementary skill sets represented on the team.
- **Start out strong.** Arrange initial commitments from your circles with the goal of securing 30% funding on the start date. Campaigns that start out with 30% of their goal met are much more likely to succeed and seem appealing to new donors.
- **Play the field.** Identify your outer circle and potential new contacts. Never stop reaching out or making new connections.

You can't do it alone! Reach out and take the help and talent that you can access.

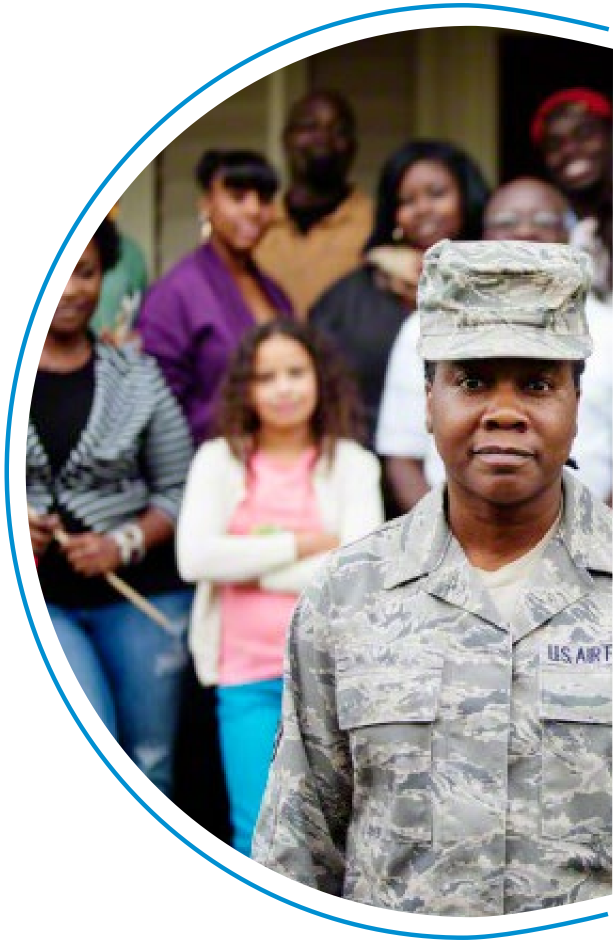

How to Assemble Your Team

How to Coach Your Crowdfunding Team

Build a team. Crowdfunding works because people working together can make things happen. Be sure you have the right team for your crowdfunding campaign. Successful crowdfunding campaigns really depend on the commitment of the team.

Who Are They?

Identify your core team. Successful crowdfunding campaigns have a committed team. Before the beginning of the campaign, if possible, get at least five close friends or family members together to help. These should be people that you know you can count on to help you get something done. They will stick with the campaign no matter what. This is your core team. Talk as a group.

They understand crowdfunding. Have planning meetings with your team and discuss the campaign as a group. First, explain crowdfunding so that your team has an understanding of the basics. This ensures their feedback and brainstorming is more useful.

They know the story. Let your team work on messaging; they know the story. Start writing down the compelling aspects of the story and consider asking trusted friends and family for feedback as you go.

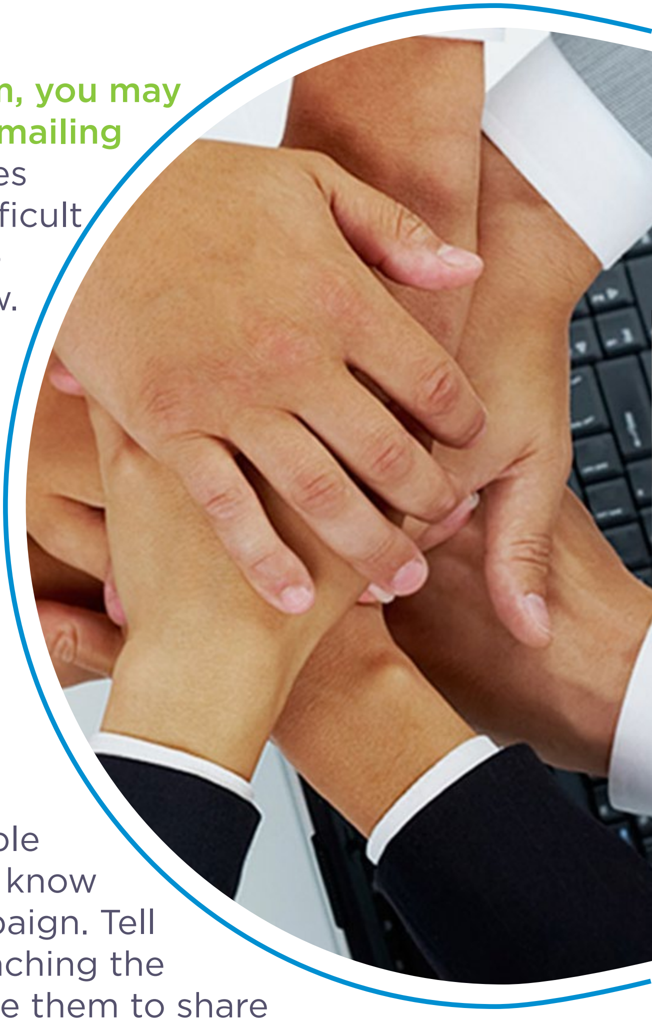

When you gather a team, you may gain a potentially huge mailing list of people. Many times when people are in a difficult situation, others want to help but don't know how. Let your network know that just being involved and spreading the word is something they can do to make a difference.

Get your communities engaged before the campaign. Tell them, "This is what we are doing, do you want to help us? Will you spread the word?" Prewire people ahead of time. Let them know you are planning a campaign. Tell people when you're launching the campaign and encourage them to share the information with their friends.

Share Connections to Create Your Peripheral Team

Ask those five team members what ideas they have and if they know more contacts. Think and discuss how you are going to manage this as a group. Have each person think about their contacts and connections.

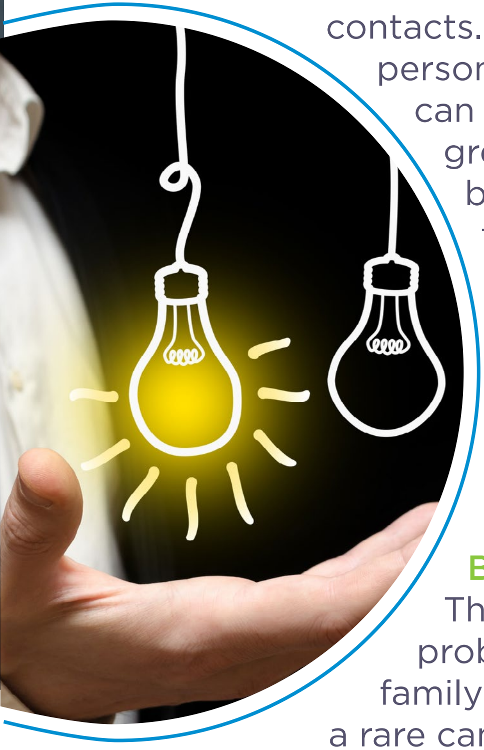

What Are Their Skills? Team members will have different strengths based on their skills and contacts. Play to their strengths. One person may have media contacts they can reach out to. Another may be a great communicator and want to be responsible for answering all the campaign emails. Another may have experience with offline fundraisers like chairing benefits, organizing a car wash to raise money or connecting with the high school PTA. Make use of your group talent and strengths.

Be creative with what they have. Think creatively about solving the problem as a group. For example, a family from Kentucky has a child with a rare cancer. They have a limited network of family and friends, but they gathered a local group of people at the gym who Zumba to raise money for the child. Publicize any campaign efforts in the community and on your fundraising page. Get local merchants involved. Let them know if it is a tax deduction. Name recognition and being listed as a contributor to the effort is important to many donors.

They communicate. Your team has a huge, collective mailing list of people. Even the most peripheral members can help by spreading the word. Your team must actively promote the campaign. Also, encourage everyone on the team to get involved with their own list to secure donations.

Raise funds as individuals and team members. Crowdfunding is a testament to the power of friend to friend fundraising. Encourage everyone on the team to get involved with their own list to secure donations. The team should be willing to work together on all aspects of the campaign including creating a video to support the campaign that they will share with their connections. They need to be committed to reaching the goal. They must create and follow the plan together. The campaign has to reach and

engage people, and this is most possible with a concerted group effort. Successful campaigns raise most of their money from first- and second-degree circles before they get bigger.

Find your community experts. Assign one team member to search for a smaller or startup PR (public relations) company in your area and try to get them involved. It will be easier for them to gain traction and visibility. The benefits to them are that they look good for supporting an important cause, and that they can use a successful campaign as an example of their work. Find other local "experts" who can help you, from media contacts who will publicize the event to artists and business owners who are interested and may donate goods and services.

They fit in the team's organization. Think and discuss how you are going to manage this as a group. Have each person maneuver their network and connections appropriately to fit the plan. Jamil Newirth's campaign team created a poster with his picture on it that they used for every benefit and party. They used the picture on all the social media sites to talk about the upcoming events, parties and the campaign. They got several businesses in the community involved in the campaign to raise money. The team also had one person with connections to media. Thanks to all of these connections, the campaign's parties and benefits were in the news, on the radio, and on TV. At the parties, they had artists and raffles, and one party raised $25,000. One of Jamil's hobbies is surfing and connecting with that community really helped support him and his campaign efforts.

Case Study: Successful Team

Jamil Newirth just finished law school and planned to study for the bar exam all summer before he started his new job in August. Excited about his future, he planned to spend every day that summer in the library preparing for his test. Suddenly, he began having excruciating headaches that sent him to the emergency room. After a battery of tests, he learned that his headaches were being caused from stage 4 Glioblastoma Multiforme, a cancerous, fast growing brain tumor with a high rate of recurrence, close to 100%.

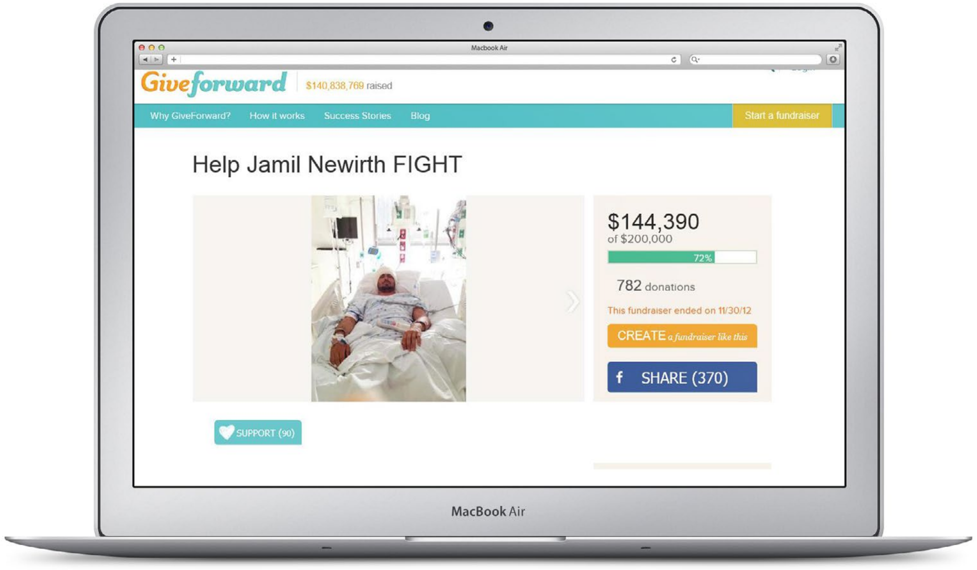

In addition to this news, when he graduated law school in June, he let his insurance lapse for the two months in the summer. He had a job starting in August and figured he would be at the library studying every day. Young and seemingly healthy at the time, he thought if he got a cold, he would just go to the clinic.

After the initial shock of the diagnosis, Jamil and his very close knit group of friends from his community in Hawaii came together to help him. They formed a team to help him raise money. They started a crowdfunding campaign on Giveforward. They made a poster with the picture on it and it went all over social media. Their efforts were online and offline. They began having parties to raise money for the cause. The poster went to all the parties. People posted on Instagram about the parties. The team reached out to several businesses including one that put the poster on t-shirts for men and women. Someone in the group was starting a small PR, public relations, firm and made extraordinary efforts to reach the media. The poster was used for press releases too. Jamil's campaign on GiveForward raised $144,390.

Team Checklist:

- ☑ Gather close family and friends
- ☑ Brainstorm and plan
- ☑ Assign tasks- online and offline
- ☑ Share connections
- ☑ Publicize and promote on all team member's social media sites

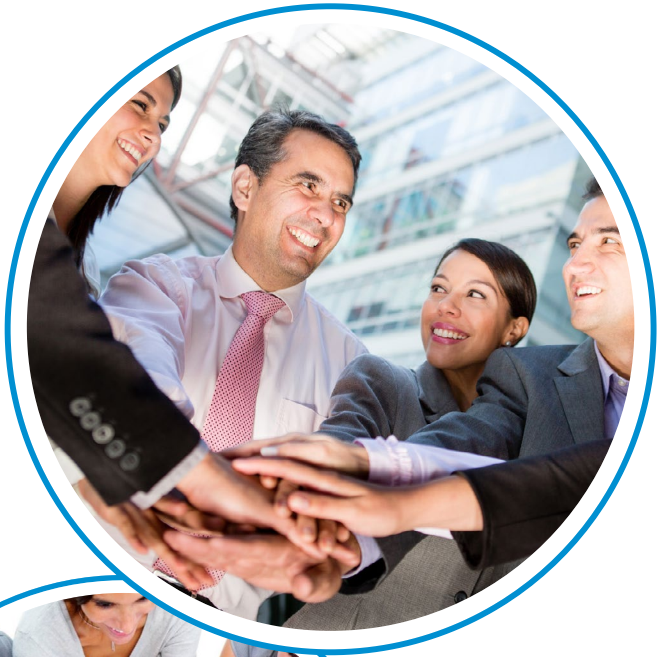

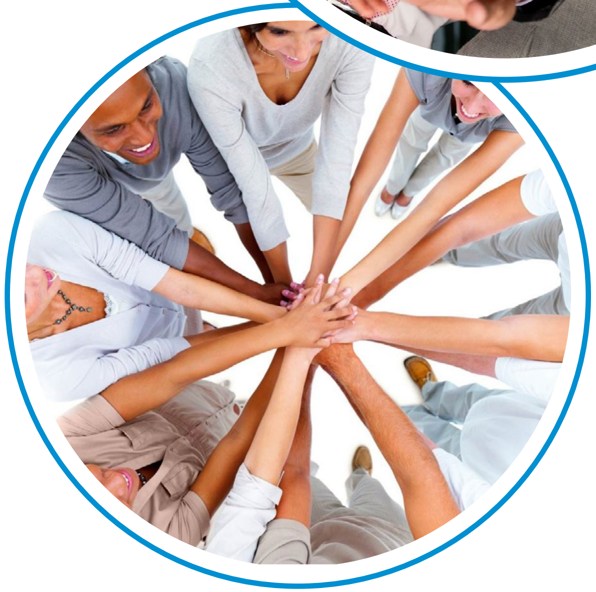

Write an Awesome Crowdfunding Email

Rare Genomics Institute

WRITE AN AWESOME CROWDFUNDING EMAIL

How to Write a Compelling Email

Time it right. You want to know that you have raised the first third of your fundraising goal before the campaign begins. The key to this is to get the commitment before the campaign begins from your network. Emailing your network before the campaign begins asking for their support is the best way to get your community engaged.

> **"When you compare crowdfunding to fundraising offline, you see that giving is not a rational thing. At end of the day, you are appealing to emotions. There is an important human element since there is not an exchange of goods with crowdfunding."**
> **—Devin Thorpe**

On the day of the launch, you want everyone to see that you have those funds raised. Some people may be skeptical of online fundraising efforts; psychologically they will feel more comfortable to see that others are donating to this important cause. On your launch day, if you have a significant amount in donations from all of your work before the day of the launch, it signals that you have a great cause that people should support and that the cause is legitimate. One of the most important aspects to the campaign is to email potential donors. The whole campaign hinges on your messaging before you launch the campaign.

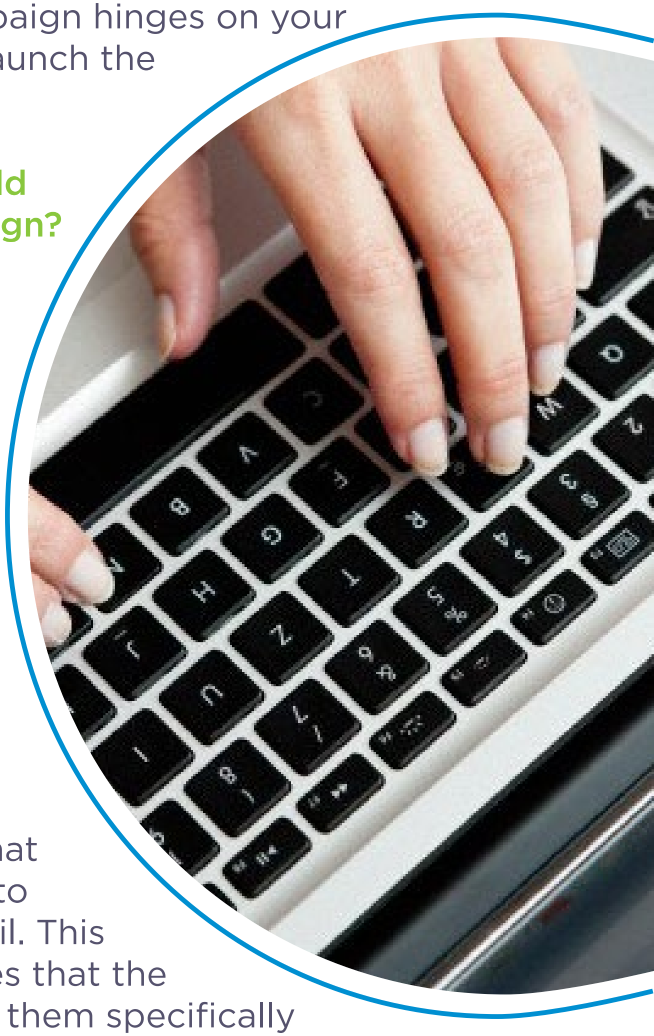

How many people should send emails in a campaign? Assemble your email team. Crowdfunding expert Devin Thorpe recommends that 10 people on your team should send out emails to their networks.

Crafting the message. What should the email say? Each message should be personalized, so use your contacts' names in the first line along with something that shows your connection to the recipient of the email. This way the recipient realizes that the message is intended for them specifically and isn't a mass mailing. Personalized emails are a

lot of work, but they offer a higher probability of success.

Tell the story. In the message itself, continue this personal approach: “I wanted to tell you about a friend of mine and their child.” Write an email that tugs at the heartstrings and signals that you really care about the campaign. Talk about the child or individual the campaign is for, their family, their illness and the imminent need to find some answers.

Give important information. Talk about the potential of the research that hopefully can change lives.

Don’t forget to ask. Finally, tell them about the cause and ask them to donate when your campaign begins. Try to get a commitment for them to support the campaign by donating on the launch day of the campaign.

Crowdfunding expert, Devin Thorpe recommends following this exercise to write the email:

How to Write Your Message

Dear Mom. Sit down and write an email to your mother about why she should give her hard earned and saved money to help this family. You can’t use marketing language with your mother. She will know if you are exaggerating. Remember it’s your mother, so make it sincere.

Best Friend. Write a similar email to your best friend and tell her why she should support this family.

Craft the Final Message. Now, you have ideal, authentic language for your message. This is very different than an email that a marketing department would write. You can craft these personalized emails into a template for reaching out to hundreds of other people.

Who Gets The Message?

First wave. Send out an email to all the people in you close circle. They need to immediately realize this is not a mass email to every one of your contacts. This personalization will greatly increase

your chances for donations. Using this approach, you can get a much higher level of participation to reach your campaign goal.

Second wave. Your core team sends out an email to all the people in their close circles.

Stay organized. Keep a log of the emails you have sent. Write down every commitment to support your campaign when it begins. For those that commit to a donation, send another email out on the launch day of the campaign. This follow up message will first remind them of the mission of the campaign, then thank them for pledging to support this cause and finally end with the exact link to the campaign's donation page, not just the homepage of the crowdfunding website. Potential donors are busy, and they may be viewing the email from smart phones. They will lose interest if it is not easy, and if they have to go through multiple steps to get to the correct page.

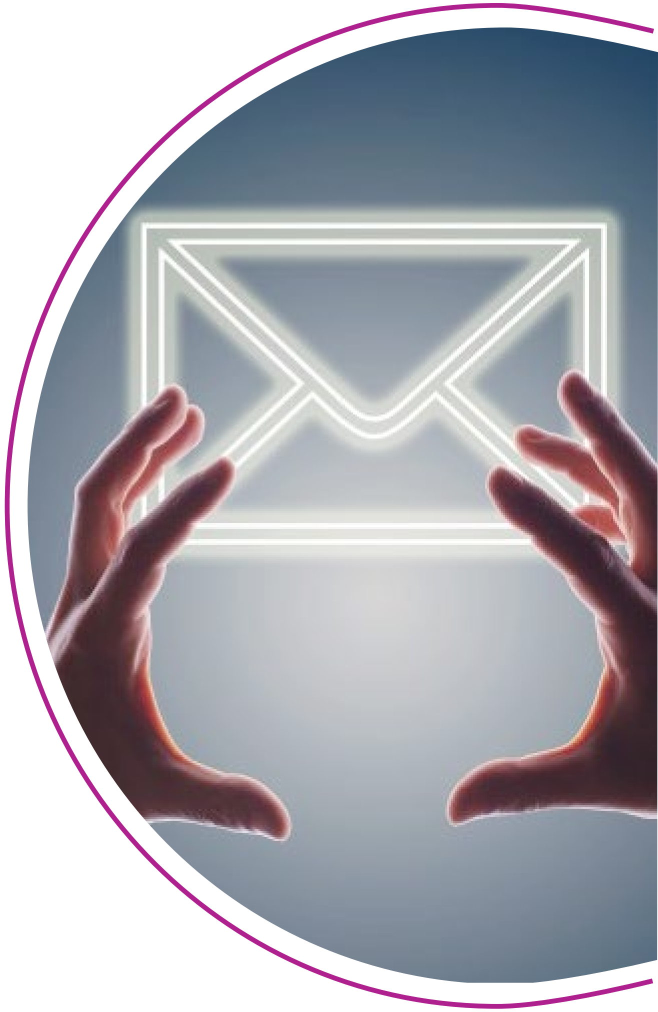

Email Checklist:

- ☑ Personalize the emails.
- ☑ Giving is not rational, so make a heartfelt appeal.
- ☑ Write an email to convince your mother to support the campaign.
- ☑ Write another email telling your best friend about your campaign.
- ☑ Put links to the exact fundraising page so it is easy to connect

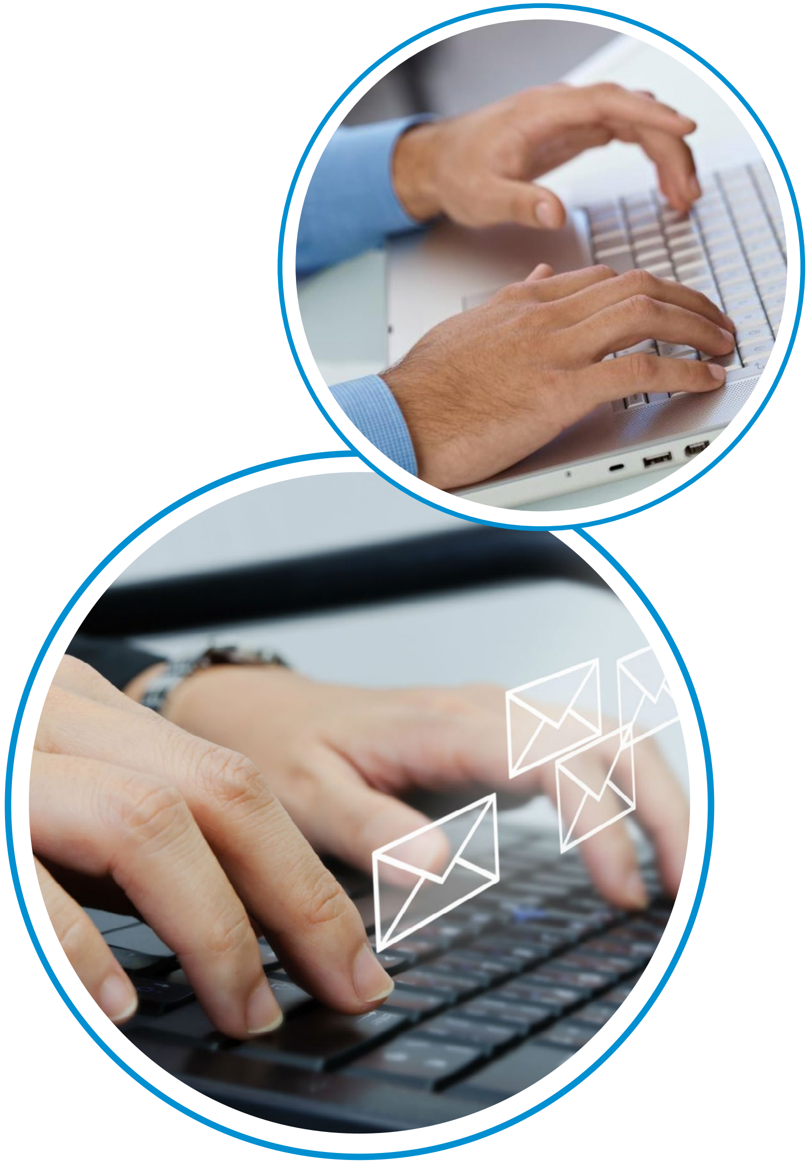

Phone Calls are Important

Rare
Genomics
Institute

Phone Calls are Important

Reach out to your top 20. In addition to sending a very personalized email before the campaign begins, Devin Thorpe recommends that you call the top 20 to 30 people on your list personally in the 30 days before the start date. Explain what you are going to do with the campaign and let them know when it is scheduled to begin. Ask them to help. Let them know it's okay if they can't donate, but still tell them what you are doing. They can still help by telling their network about the campaign.

Pull on people's emotional heartstrings. Tell them why you need the money, not just how much you need. If you need money for an illness, explain, "I need the money for a medical procedure," and then talk about what it is. In simple terms tell them how you will use the money.

Make it one call per day so it is not overwhelming for you. Call people you know would do anything they could to help you. Once you get a commitment, tell them you will send them the link the day the campaign starts, so they can make their donation.

Luke Miner, co-founder of YouCaring, encourages people to share the personal aspects of the campaign. He explains that people that are successful in sales connect with others. Similarly, in crowdfunding you have only a few minutes to connect with someone and have them empathize with the situation. It is this connection that prompts people to donate to the cause.

Connection rings. Everyone has multiple rings of connections. First is the ring of your inner circle of family and friends, followed by the next circle of your co-workers, associations, and organizations. YouCaring recommends reaching out to people in all of your rings of connections.

Phone Calls

- Personally call the top 20-30 people before the campaign begins.
- Make one call per day the month prior so it's manageable.
- Tell them why it's important to reach your goal.

Goal Setting for Fundraising

Rare
Genomics
Institute

Goal Setting for Fundraising

Set clear, realistic, measureable fundraising goals and timeframe. Know what amount you need to take care of your costs. You can have a "reach" goal too, but people often like to fund projects that demonstrate success and confidence. On the web page, state the goal, along with the amount and percentage raised to date. You can also set a new goal after the first is attained. When a campaign has statistics showing the initial goal was reached and exceeded, more donors will have the confidence to add funds to an already successful project.

When do I need the funding? Early goal setting is important. You do not want to do half a campaign. It allows you to plan and know that you have enough money to cover all of your costs. You must also decide on a goal in order to evaluate which campaign type of "All or Nothing" or " Keep it All" is best for your cause.

Urgency toward the end. For research based projects, you can set incremental goals. And while different campaigns have different needs, crowdfunding experts agree that you need a time limit. This creates urgency and moves people to act and donate.

How much do I need? Be sure to explain the costs of the elements of the campaign and show where the money is going. Let donors know how their money will be used, with specific examples if you can. You can say that $50 pays for this important test that the child needs and then name the test. This detail makes it easier for people to understand and empathize. This in turn makes them more likely to donate to the campaign.

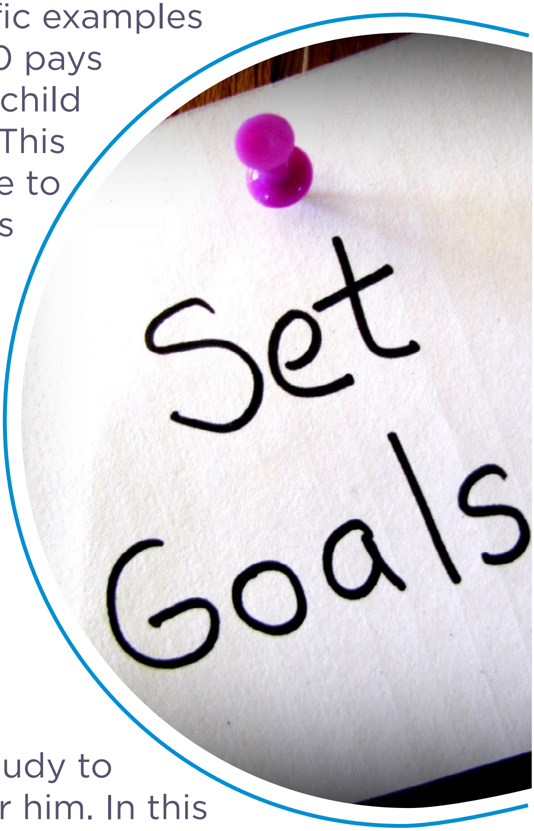

When Zsuzsanna Darvai wrote about the campaign for her son, RG patient Balazs, she explained: "The only goal is the cost of sequencing." She wanted to make sure she explained that insurance would not cover this. She also told everyone that Balazs was lucky enough to be included in the study to try to finally find a diagnosis for him. In this way Zsusanna explained that her donors were helping to find a diagnosis, "Your help to raise the money to find answers for him."

Jeneva Stone started a campaign to raise money for the costs of DNA sequencing of her son; her goal was to find a diagnosis for her son who had an undiagnosed rare disease. A reporter asked her, “We are in a recession, how do you think you are going to accomplish your fundraising goal?” Jeneva answered by framing the need in small increments: “I will tell my audience that if I get 750 people to each give me $10, I can do it.”

How to Measure the Impact

Talk about the impact. Let the audience of potential donors know that their gift of money can change the child’s life and maybe have an even bigger impact. Remind them that if the money raised is for a child’s clinical trial and helps that child, their donation could also impact many other children or people waiting for answers.

What’s in the future? It could potentially be a solution, a new treatment. This would be rewarding to donors knowing that they helped fund and make a difference in many lives, and that they have contributed to general scientific knowledge.

Goals Checklist:

- ☑ Set realistic goals.
- ☑ Frame it in small increments.
- ☑ Explain how to reach your goal.
- ☑ Set a time limit for the end of the campaign.
- ☑ Create urgency.
- ☑ Talk about the impact.

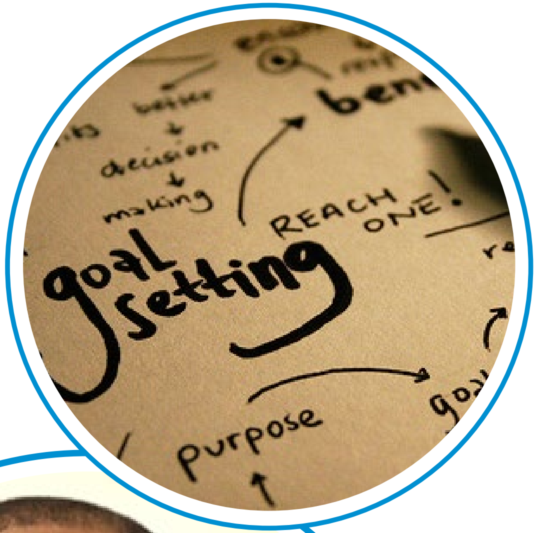

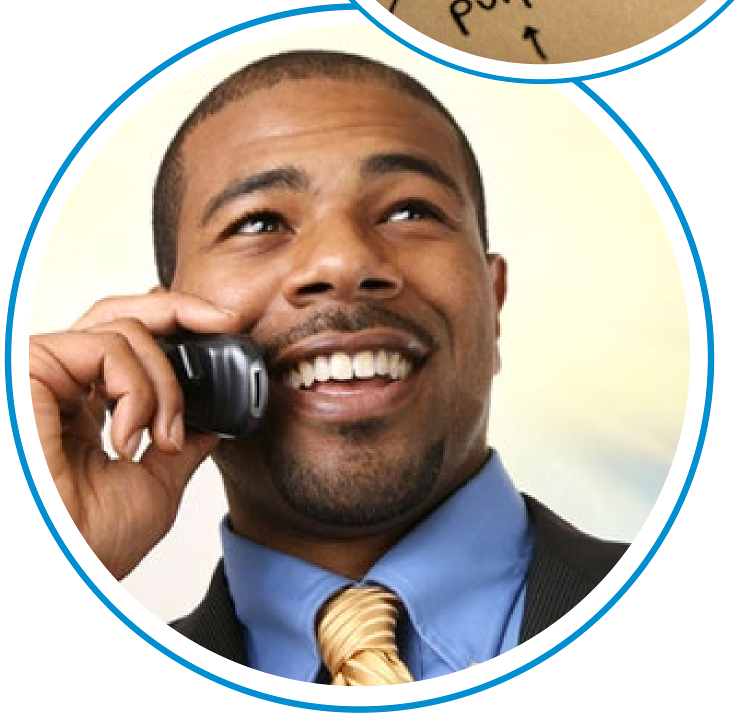

How to Make a Great Crowdfunding Video

Rare Genomics Institute

How to Make a Great Crowdfunding Video

What Does The Video Say?

A Personal Story. If the campaign is for a child's medical costs, a video of the mom, dad or a close friend telling the story of the child's illness can be impactful. If someone outside the immediate family is running the campaign, this outside voice can help establish more credibility. You are creating an immediate, personal endorsement. Crowdfunding expert Devin Thorpe explains, the video voice should send the message that "I personally know their situation, and I know they need help."

Focus on the individual. After you organize your team, create a video with the sick person as a primary focus of the video. Ideally you will show a brief yet poignant "snapshot" of the way this child's life is—one that shows the viewer the problem in a compelling way so they will want to help. If the medical campaign is for a child, and the child is in the position to actually speak and explain their situation, that may also prompt deep empathy in others.

Be creative. You're not educating a classroom about the disease; you're making connections with people. Share the details. Explain the specifics of the disease. The video must provide a stark, emotional portrait of the problem.

Be sincere. You do not have to have a professional video. However, in order to make an effective video, you do have to build trust. A video helps you reach and connect emotionally with more people than does a simple webpage. Potential donors can read about someone's problem, but if they can also see and hear how it is affecting them, they are more likely to connect and feel empathy. It does not have to be a high tech video, but it should show the problem or need and how you will solve it with their help.

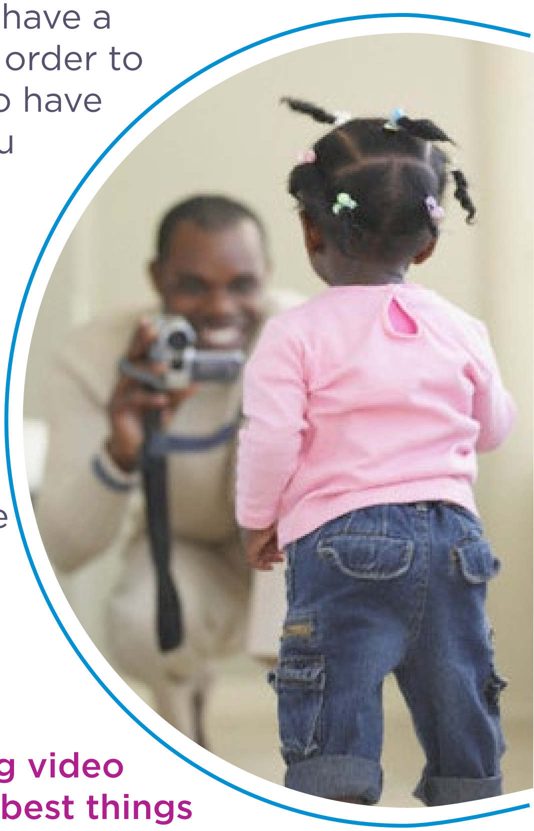

"Getting a crowdfunding video to go viral is one of the best things that can happen to a campaign."

Inspire donors. Offer a way to help: "But there is hope." Then tell them why. In this way the video

and campaign both connect with viewers and offer an actionable step they can take- something they can do with this new found connection.

Think ahead. Plan to eventually transition from the initial short term goal to longer term goals, and make your video work not alone, but as part of a series.

Technical Issues

You don't need a professional video. An effective video builds trust and helps you reach and connect emotionally with people. It does not have to be a high tech video, but it should show the problem or need and how you will solve it with their help.

The video should be no longer than three minutes. In the first 10 seconds-describe what the campaign is and why funding it is unique. If putting a link to a video in an email, always say in the email the length of the video and state that it is a video.

You may be able to get professional help. Although your video does not have to be professionally made, you may well be able to find a professional videographer to help your campaign. Even if you can't get someone to make the video, they might be willing to listen to your story and tell you how you should go about making it.

Use the right language. Don't ask people to donate at first. Ask them for three minutes of their time to watch and share the video. They need to know it's short and worth the time. After they watch, they can decide whether or not to donate (and if it's well-made, they usually will).

Make it easy. Show the direct link to your campaign. This keeps it personal, and easy.

Focus on the bigger picture. Align yourself with the larger crowd effort. This campaign is not just us, it's all the donors in all the countries.

Use social media. Create a hashtag for everything you do. Once you have media contacts, give those updates. Share something on social media every day.

Aim for viral success. Make sure it's emotionally rich and focused on the person and issue at stake.

Case Study: Viral Video

One of the most successful fundraising campaigns was “Saving Eliza.” The case study tells the story of how Eliza’s dad, Glenn O’Neill, reached out to create a viral video and successfully raised over $1.8 million. The funding is for a clinical trial that he hopes will stop Sanfilippo Syndrome and save his daughter Eliza’s life.

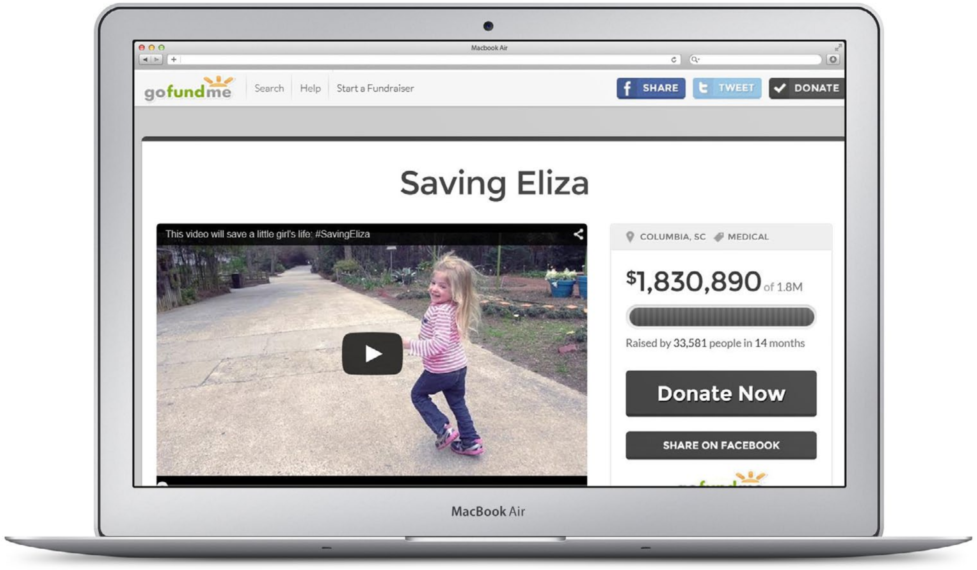

In July of 2013, Glenn and Cara O’Neill experienced every parent’s worst nightmare, doctors informed them their 4 year old daughter Eliza had Sanfilippo Syndrome-Type A. This rare, fatal disorder affects about 1 in 70,000 children born.

She is happy and playful now, creating a moving contrast with what is to come. “Looking at her, you would not be able to tell, but that will all change,” Glenn said. Sanfilippo Syndrome-Type A, will dramatically alter Eliza’s life. By 6 years old, most children with this syndrome have irreversible brain damage affecting their ability to walk, talk, and feed themselves. Children with Sanfilippo Syndrome are missing a necessary enzyme. Without it, they develop toxic material throughout their bodies and many die before they become teenagers. Explaining these issues, the video and campaign provided a stark, emotional portrait of the problem.

Next, the video inspired donors, offering a way to help. Glenn said, “But there is hope. There is a gene therapy that has been shown to stop the disease in animals. It is hard to say you are fortunate when your child has a rare disease, but we are fortunate that we have a defined hospital, Nationwide Children’s Hospital, going from animal

to human stage with a drug that could stop the disease. We still need FDA approval but what we really need is money for the clinical trials." The money they need to fund the trial is $1.8 million. In this way the video and campaign both connect with viewers and offer an actionable step they can take-something they can do with this new found connection.

Even if they raise the $1.8 million needed for the clinical trial for the possible life-saving treatment, there are no guarantees that Eliza will actually get into the trial. She may not be a good candidate for these carefully controlled clinical trials. But this is a chance the O'Neills are willing to take, and because they are, so are their donors. When people ask Glenn how he feels about raising money for a clinical trial that his own daughter may not be able to benefit from, he replies, "If a clinical trial does not happen, I can guarantee she has no chance of life."

To raise money the first thing they did was they created the Cure Sanfilippo Foundation, a 501(c) 3. The foundation was set up to stop the disease in all children. With no paid employees and no personal expenses, they were set on raising funds. "We went at it from the foundation standpoint. Tell everybody you know," Glenn said. They held fundraisers, parties, and 5K runs, everything they could think of to raise money.

Early on, the O'Neills knew that the clinical trial would need $2.5 million dollars to fund the 20 children necessary for the clinical trial, the hospital, and the drugs. Glenn said, "We found out that nobody pays for this in rare diseases. It's been basically parent raised fundraising. Sometimes, I get sucked into this false sense of hope. I was hoping that with doing all these parties, we would hit that one person that knows Bill Gates. It took me about 5 months to realize that was not going to happen. In about 6 months, we raised about $250,000, but this was nowhere near the money we are needing."

It is this despair that catapulted the O'Neills into a larger project. "In October of 2013, I created a home video of Eliza and put it on the GoFundMe crowdfunding site, just like videos I saw other families did," said Glenn. The October 2013 video raised about $40,000 of the $250,000. Glenn said, "It did ok, but it was out of sheer desperation and frustration that the idea came about. I thought, 'What was the strength we had?' Of all the people I've talked to, nobody had ever heard of Sanfilippo

Syndrome, but when I told them about it they would say, ‘You are telling me your kid is normal until about 3 then fades away.’ Everyone could not believe it- and then they wanted to help.”

Glenn thought, “Is there a way we can get everybody to know about this. We are just not spreading the story enough. I thought, ‘Could we do this?’ I thought about a viral video. It was close to midnight the night of the Super Bowl, and I went on Google and searched ‘How to make a viral video’ and wrote the first person that came up: Karen Chang, a total stranger.”

Karen Cheng wrote about 10 ways to make a video go viral. Glenn said, “ I thought I’d email her, tell her our story and ask for her advice.” Glenn emailed her and explained their story and asked her, “Do you have any idea how given our story, you would make a viral video?” She contacted people around the world about their story, and one guy answered. His name is Benjamin Von Won, and he said he had a break in his schedule and would give it a shot. “I can be at your house for a week if I can sleep on your couch,” Von Won said.

After many late night email exchanges, Glenn told Cara, “Guess what? There are 30 people coming to our house.” Glenn explains, “They stayed for 8 days, all pro bono. They took 40 hours of footage of our lives, the kids playing and interviews of us.”

Meanwhile, the O’Neills made the most of connections and worked to publicize the campaign. “I did a lot of work sending emails to the media,” Glenn said. Glenn crafted a press release about a dad trying to save his daughter’s life with a video and it worked to interest the media. A local reporter crafted a media story about a viral video in the making. The crew left the O’Neill’s house on March 30 and then on April 2, the video they created went live on the crowdfunding site. It raised $1 million in two months.

Every campaign has highs, lows and slow downs. “When it comes down to it, you do not want to lose the momentum,” Glenn says, “The challenges of any campaign like this one involve competing for space and attention in a social media world flooded with great causes.” So, how do you stand out? “The way we stood out was the video and getting the attention. The emotions of the video and call to action moved people. “We never got the big donor, that one check for $250,000. The people with a ton of money are asked by so many.

The challenge is keeping it going when it slows down," said Glenn.

Other hurdles include transitioning from the initial short term goal to longer term goals. "Everyone wants to know how much money you need to kill this disease. We are in it for the long haul. There are no guarantees that the clinical trial will work. We need funding to continue the foundation. We are also looking into newborn screening," said Glenn.

"It was the media that broke this thing and made it viral. I have 12,000 followers now on Facebook. People are engaged with the story and our updates about how Eliza is doing. We keep telling our story and getting people engaged so when it is time to ask again hopefully they will give," Glenn said.

Glenn advises, "Don't say donate. Instead ask, 'Do you have three minutes? Watch and share SavingEliza.com.' You are asking only for their time and they can decide if it is worth it to donate," he suggests. It is important to tell people how long the video is so they don't think they are getting into watching a full documentary. The video has 400,000 views as of now, and there are things that go viral on Instagram that are 15 seconds long."

> **"If there is anything we did that could possibly work for other people, we want to help"**
> **—Glenn O'Neill**

Before this, rare diseases did not come into my life, and I never knew how things worked behind the scenes. We need something that educates people as to why families like us are trying to raise the money themselves," he said. "The majority of the money we raised went to the drug. The drug took six months to make so our first batch of the drug will be given to all the 20 children in the trial."

As Eliza's 5th birthday approached, her family asked for a birthday present for her. They asked everyone to donate in a final push to reach their goal. "Give her the gift of life for her birthday," Glenn asked. The result of this effort was that The Saving Eliza crowdfunding campaign reached and exceeded its goal. In 14 months, $1,830,790 was raised from the $1.8 million goal. The gift is from a total of 33,574 people.

Takeaways:

1. **There are a lot of talented, caring people on the Internet.** You may be surprised at what you get when you are in a tough spot and ask for help.
2. **Be creative.** "The regular video of the scientific end of what happens in the brain in an illness will not resonate with many people," Glenn said. "Instead, you want to have something that gets shared and the awareness will come. Our focus was on Eliza."
3. **Be genuine.** "We trusted Ben Von Won to give us a compelling story from what he had seen in the 8 days he was with our family. It was emotional, true and genuine. It was genuine feelings. He showed the urgency even with the music."
4. **Show the direct link to your campaign.** This keeps it personal, and easy.
5. **Focus on the bigger picture.** How do you raise awareness that there are these rare, critical illnesses? We are in the situation of transitioning from Saving Eliza to the foundation's continued efforts. The important point that was made is that we need to fund all these rare diseases.
6. **Align yourself with the larger crowd effect.** "It is not GoFundMe for our campaign. It should be thought of as GoFundUs. We are using the site raising funds to stop a disease. We have 25,000 donors on the site in 70 countries. Every update goes to all of those donors." Harness the power of social media. "We hashtag Saving Eliza on everything we do. Once you have media contacts, give those updates."

   In one day, we raised $125,000 on the site, so we told the media we are about to break a record on GoFundMe. Social media translates to fundraising because of the ability to share. With a blog, people can read it and click share. Every time they did it, the video is embedded. Glenn said, "Many people told their network of friends, 'You have to watch this video to understand this family's story and understand this disease.' On Twitter you can tweet anyone."
7. **Learn to build empathy.** "You have to know the strengths of the disease or your cause and how to reach people to make them aware of it. When I talk with someone for the first time, I think about their reaction to hearing about Sanfilippo Syndrome. That is the audience

you want to reach. It is the people that have never heard about rare diseases or Sanfilippo Syndrome. Have them put themselves in your shoes. Many will then say, 'I cannot believe a disease like this exists, and they will want to contribute to help you stop it."

8. **Improve your skills.** Glenn O'Neill recommends the book *Jab, Jab, Jab, Right Hook* by Gary Vaynerchuk. It explains how to best use social media, provides good examples of Facebook and Twitter posts, and gives good advice on how to explain your story and to involve others in your cause.

Video Checklist:

- ☑ The video should be no longer than three minutes.
  - ☑ In the first 10 seconds-describe what the campaign is and why funding it is unique.
  - ☑ Next, tell the audience who is receiving the funding. People like to know the personal aspects of the story.
  - ☑ Tell the history behind the organization or illness. People want to know it is a legitimate cause, problem, or organization.
- ☑ If putting a link to a video in an email, always say in the email the length of the video and state that it is a video.
- ☑ The video should make a connection.
- ☑ Engage others with the video. Try to make it feel as if it's you talking directly to them.
- ☑ Put links to the video with any social media.
- ☑ Videos can significantly increase donations.
- ☑ Does NOT have to be a professional video.
- ☑ Sharing the video can get the message out to a wide audience faster.

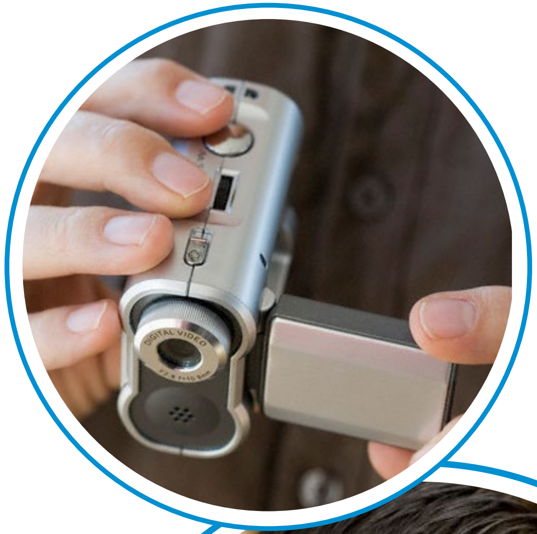

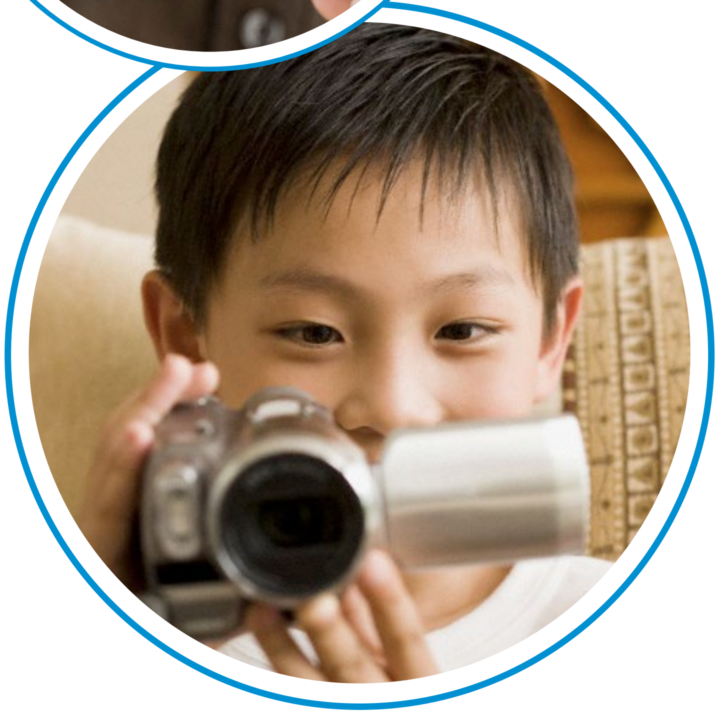

What's a Great Picture?

Rare
Genomics
Institute

WHAT'S A GREAT PICTURE?

Get the picture. Make sure you have many high-quality photos for use on your site and on social media. As we discussed, videos are very influential and help you share your message. Pictures are also important and help personalize and differentiate your campaign. Cute pictures of kids will help you connect with your audience. You want to have anywhere from 3 to 10 emotionally gripping pictures on your campaign page. This shows others what the patient is going through. Include pictures of a hospital bed or anything that helps convey the situation as well as pictures that are smiling and hopeful.

Make the donors feel they are part of the project. Pictures can help them see your life from your perspective. For children battling a rare disease that is probably unfamiliar to potential donors, photos show how the disease affects the child and the family. Show them what life looks like and how the money raised will impact them in a positive way and really make a difference.

Pictures build trust. Luke Miner, co-founder of YouCaring, encourages people to share the personal aspects of the campaign. In the few minutes you have to connect with potential donors, connecting visually creates empathy. Pictures build a brand for the campaign. Research says it takes between 6 to 10 touches before you get someone to act. They often do not pay attention in the beginning.

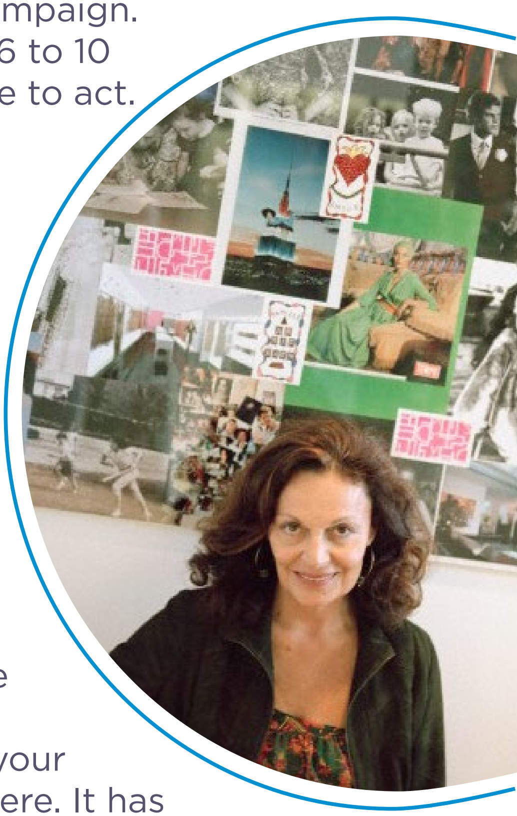

GiveCorps founder, Jamie McDonald says, "You want to build the identity of the campaign everywhere. Charity Water is a good example of this. The same image of a well in Ethiopia is used throughout their messaging. By the third time you see it, you know there is a well there, and you are touched." She recommends iconic images for campaigns too. "Come up with your iconic image and use it everywhere. It has to effectively convey what you are raising money for. There should be a core story and tag line, and then if you add video it is another touch point. If you have a fantastic photo, great, but know that it does not have to be professionally done."

Pictures Checklist:

- ☑ Gather 3-10 emotionally gripping pictures.
- ☑ Make the donors feel a part of the project.
- ☑ Come up with an iconic image and build the identity of the campaign.

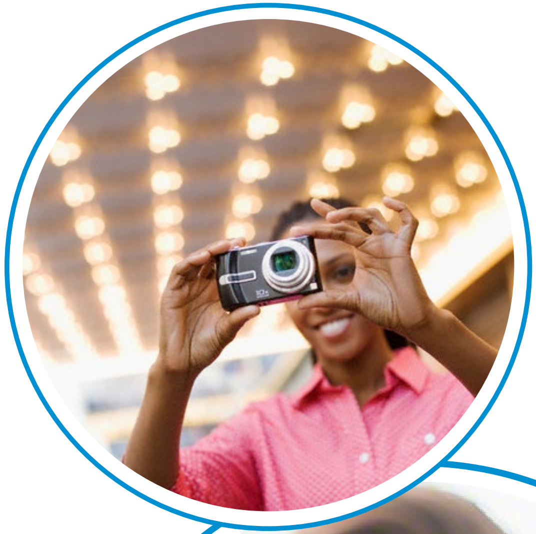

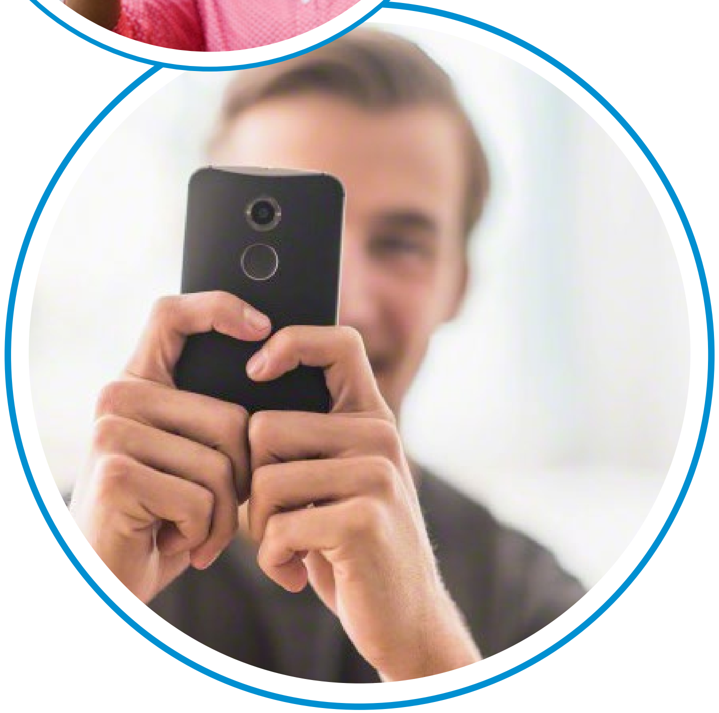

What About Perks and Gifts

Rare
Genomics
Institute

WHAT ABOUT PERKS AND GIFTS

Gifts or no gifts? Some medical campaigns do offer perks or gifts. If you decide to offer some, consider making the reward something personal. Larger monetary causes have 6 to 7 levels of gifts at most, to avoid confusion. Be creative and tie in rewards to specific donation amounts, realizing that most people donate between $25 and $50 to crowdfunding campaigns.

Levels of donations. Smaller donations could receive a personalized thank you letter. This is a great gift. If the campaign is for a child, a gift could be a framed painting done by the child. Another example could be a video thank you message or a shout out on the donor's website. Donors may want an invitation to a monthly webinar, or ongoing information on the disease if you have connections and access to researchers.

Show potential donors that long term involvement brings rewards. Experiential prizes are popular. For a team fundraising employee challenge, if the company really wants to get them involved, the top fundraiser could earn an extra vacation day or park in boss' spot for a period of time. This creates team building and motivates the employees to participate. Donations for larger monetary amounts could receive naming rights, or branding on your site.

Sharing the journey. Remember, most people giving to medical causes just want a thank you in return. You can also be creative in who says the thank you. For example, a patient advocate could say thank you. The patient could say thank you. A researcher could say thank you. With medical campaigns you are ultimately dealing with people's well being, so passion and recognition are important. Let them be on the journey with you. Show your appreciation and give updates.

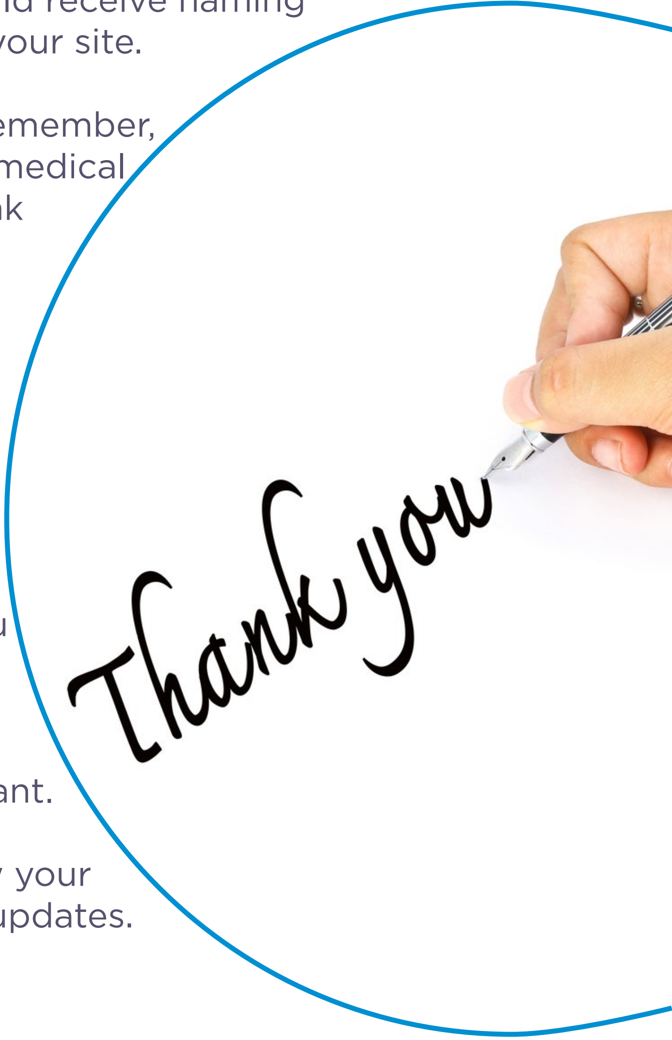

Gifts Checklist:

- ☑ Be creative.
- ☑ Give them something personal.
  - ☑ A personal thank you letter
  - ☑ A painting done by the child and framed as a thank you
- ☑ A video thank you message or a shout out on their website.

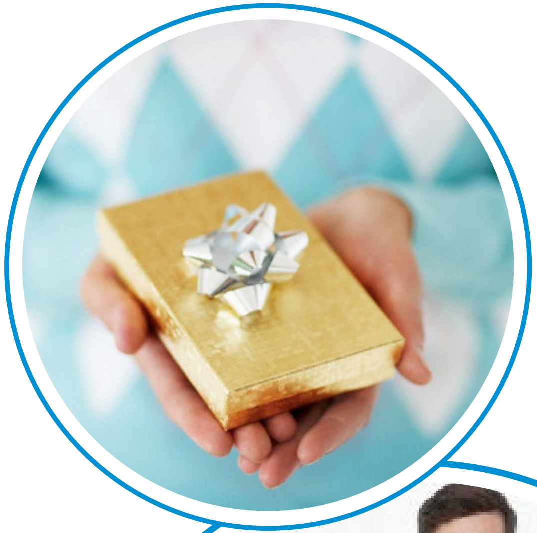

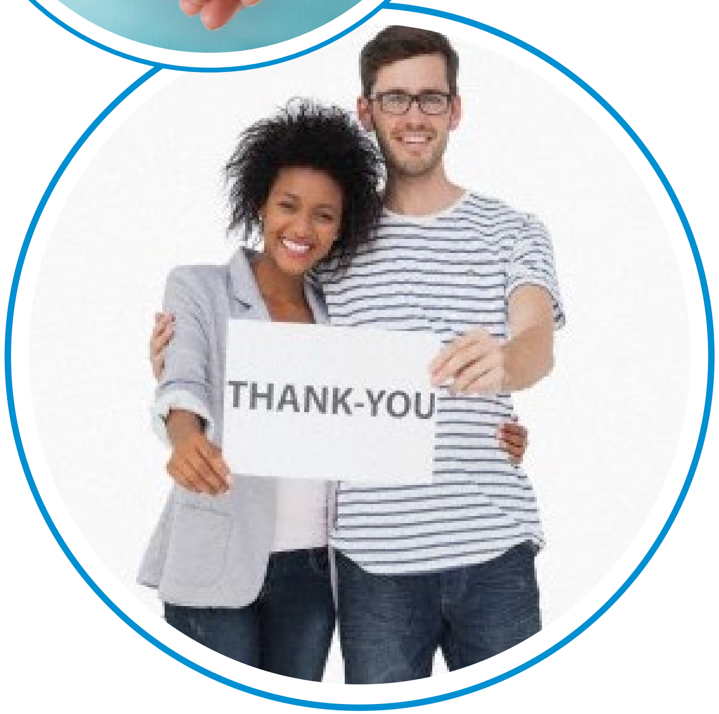

Launch Day

Rare
Genomics
Institute

LAUNCH DAY

What to do the first 24 hours?

Launch party. On the campaign launch day, experts recommend inviting people to a launch party. In the case of children with medical illnesses, this may play out differently. If the child can have visitors without the risks of getting sicker, people can come over for refreshments and an opportunity to take some pictures that they can share with their circles of friends. This will also allow them to tell the campaign story effectively and in a personalized way. The party can be low budget and in the home.

Invite many. Crowdfunding expert Devin Thorpe advises getting as many people there as you can. If there are 30 people at the launch party and each one tweets it to 1000 people, then 30,000 people now know about the story and the campaign. Partygoers can share a picture of them with the child and tell their circle, "I pledged $50, and here is a picture of me with the little girl I was telling you about," he said.

Send the email with the link when it's live. On a few platforms, you can get donors pre-pledged; research your site in advance. The crowdfunding site, Razoo, has the ability to do that. Go to your log of pre-campaign commitments. Then on launch day, send out your reminder emails. Make sure the message is no longer than 4 sentences and includes a direct link to the campaign.

Focus on the social impact of your campaign to best make use of your network. First provide an update on the campaign and its mission, and then thank them for their pledge and provide the link. Keep the email mission- focused not money-focused, and post it on all social media sites where you and your team are active and have a presence.

Make it personal. The biggest mistake people make in their campaigns is to talk about the project and not about themselves. For example, the father who has sons with a rare disease talks about his passion and his work. Be prepared with a team or team member to answer every email. Some campaigns assign one team member to sent out and answer every email so that each response is written in the same voice.

Ask and use feedback on your campaign. Put every effort into the start of your campaign, there is a velocity that happens. Sam De Brouwer believes the first 3 days are critical. Create a force that will continue with the crowd.

Case Study: Engage Your Audience

Sam De Brouwer, co-founder of Scanadu Scout, also led their successful crowdfunding campaign. The campaign raised over $1.6 million on Indiegogo with the initial goal being $100,000. Scanadu Scout is the handheld medical device that tracks your vitals.

When thinking about engagement with your audience of donors, Sam recommends, "Talking to the groups you are working with and finding what is important to them. From this, generate a list of goals, and gifts associated with those. Think of perks coming from the 'DNA of who you are.'" The Scanadu Scout campaign effectively engaged donors to get valuable feedback on their device.

You designed your messaging, set your fundraising goals, created a video and got great pictures. You reached out by email and phone to your inner circle. You have carefully planned your outreach before the campaign begins to warm up family and friends. You told your network what you are doing and when the campaign begins. You got feedback from your team. Now what?

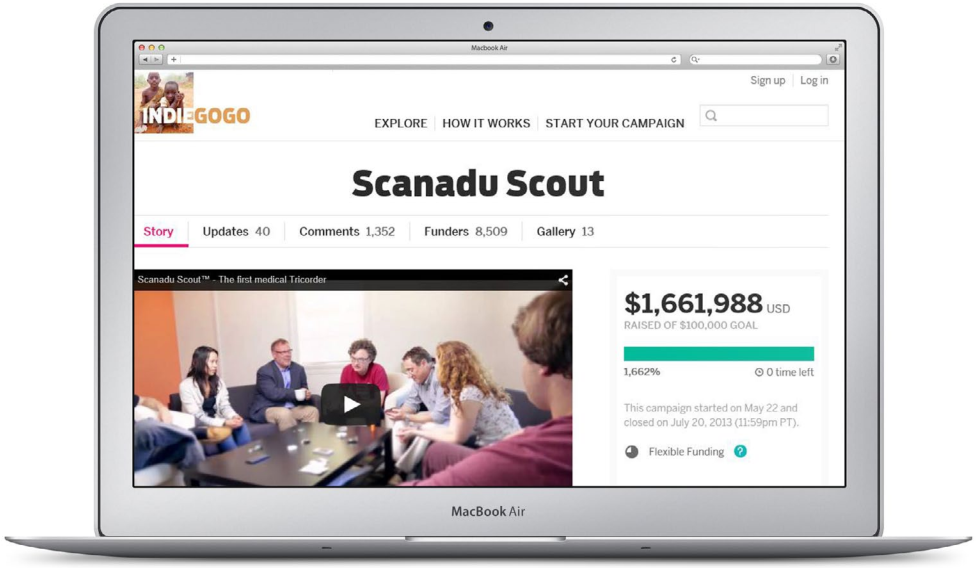

During the Campaign

- Hold a launch party on Day One
- Provide regular, detailed updates
- Use social media for the campaign every day
- Engage in offline fundraising and link it to the online work
- Contact the media
- Elicit feedback and act on it
- Make a strong final push at the end of the campaign

Launch Day Checklist:

- ☑ Host a Launch Party.
- ☑ Email all committed donors that have agreed to pledge.
- ☑ Send email blasts to contacts and answer every email.
- ☑ Check the web page and donor section.
- ☑ Post to all social media sites that you and your team are active on.
- ☑ State a call to action.
- ☑ Ask everyone to share.

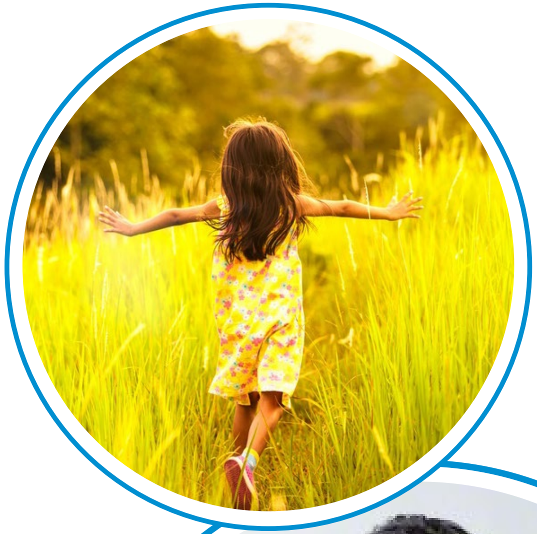

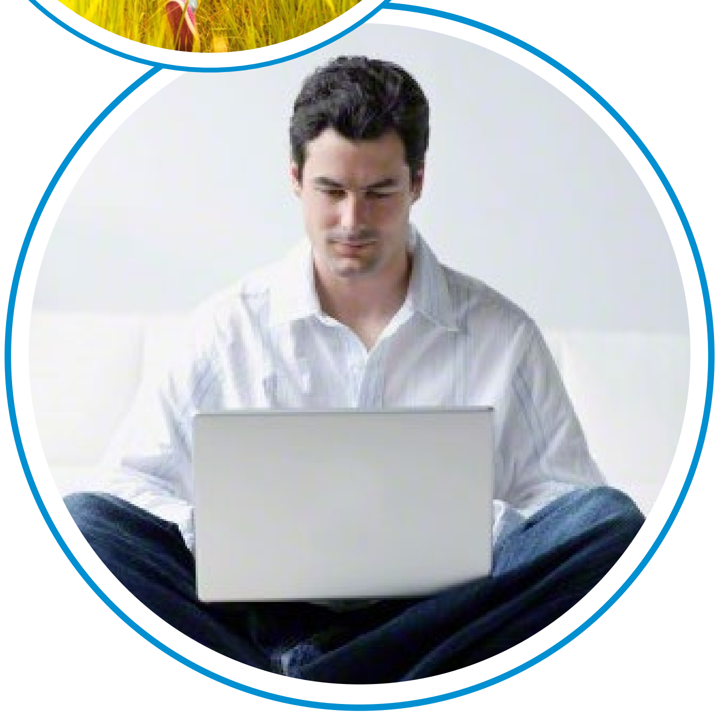

Running the Campaign

Rare
Genomics
Institute

RUNNING THE CAMPAIGN

Be flexible. Your plan can and likely will change as you are going through the crowfunding process, but the planning phase forces you to think strategically. Consano founder, Molly Lindquist, explains, "80 percent of your fundraising dollars will come from your first and second degree connections." Make sure you are connecting with them and keeping them updated. Try to make it their cause too.

Media

When you are in your campaign, always look for new ways to promote it. It is very important to reach the target audience. Blogs, email, print, social media, TV, radio and events are all areas of focus for successful campaigns.

Know how to use social media. During the campaign, actively tweet, use Facebook and Instagram. Use any social media outlets available to you and your team. Research and consider social media ads; one family successfully reached 1,500 people with one $15 ad. Always provide the direct link in every share or post. Send everyone you contact to your patient page. Give updates, add new pictures, and work to keep the audience in the campaign. They should feel like they know you. And don't forget to elicit suggestions and act on them as needed.

Update regularly so your campaign goes into the news feeds. Instead of asking people to donate, ask them to share your campaign. Facebook and Twitter are the two biggest social media sites used by most crowdfunding platforms. They are recommended because they quickly cross different platforms. Denny Luan, co-founder of Experiment, says, "Focus on the social aspect. In your message, make it more about the mission and the social impact."

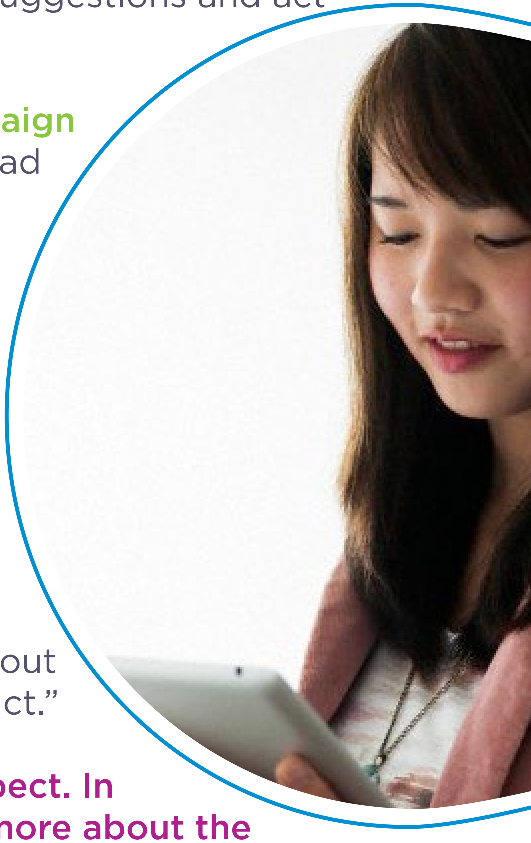

"Focus on the social aspect. In your message, make it more about the mission and the social impact."
—Denny Luan, co-founder of Experiment

Know the social good. If you can measure it, tell people what the impact is. For example, $50 would provide clean water for a family for one week. Explain how the campaign is going to help someone or something.

The media can significantly spread the word of your campaign. If you're not sure how to get the media's attention, focus your efforts to match the problem you're trying to solve. Try to get local TV stations, local newspapers and bloggers interested with an "angle" they can use for the story. For medical causes and campaigns, know the health reporters in your community. Find out if they have connection to your disease and cause. Seek out the best blogger in the space. Get them to blog about it or ask to "guest blog" to their audience. Many media outlets provide an email link for the author near their byline so writers are not hard to track down.

Expert advice. Devin Thorpe, crowdfunding expert and Forbes contributor explains, "The key to making connections with reporters is to do some homework on them. Read what they are writing. Choose writers that write about health issues if it is a medical campaign. Know the causes they care about. You can say something like, 'This is a close friend and I just wanted to tell their story about their son/ daughter's illness.'" Tell them you have read their work and that you want to share a related story with them and their readers.

Write a press release. Send a press release as well for writers that may not the time to cover the story in an in-depth way. Follow them on social media and read their work regularly.

And don't forget to follow up. Reach out to the media again after the campaign begins to tell them you raised $3,000 of the $10,000 goal and reiterate the mission of the campaign.

Zsuzsanna Darvai's campaign for her son's DNA sequencing attracted media attention and interviews on television. When pitching her son's campaign to the media, she told them that, "The reason to do an interview is to raise awareness. Genome sequencing provides new options. People need to know about genomics and the latest medical research. You have to fight for children and their needs--if the insurance won't cover it, you need to try something else."

She also explained that genome sequencing is so new. It is constantly developing, and it is one of best options. It gives people a lot of hope. She recommends going to the newspaper and

telling them this is the newest research. She also explained the work done by Rare Genomics Institute.

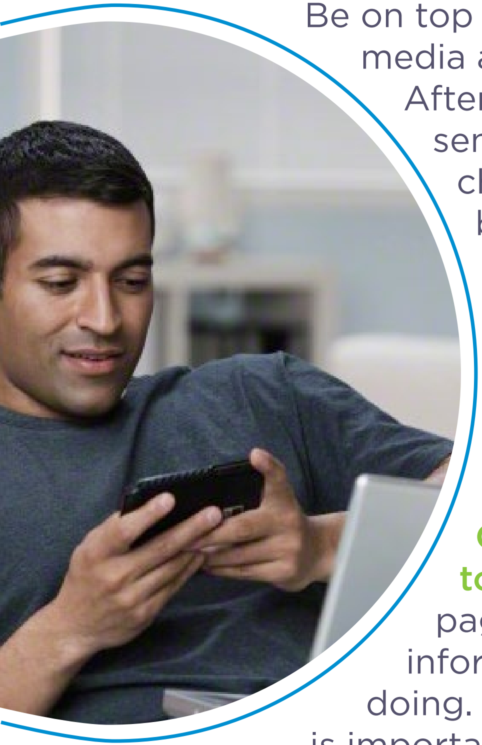

Be persistent. Don't get discouraged. Zsuzsanna said when she first pitched the story to members of the media, they did not respond. Then she went to the TV station six weeks later, gave them her flyer and said please read it. She also said maybe someone would be interested in it at the station. Be on top of it and follow up. If you do get media attention, thank that new contact. After Zsuzsanna's interview, she sent the interviewers flowers and chocolate and thanked them for bringing awareness to Balazs and other children with special needs. The TV station professionals agreed and said it was a great story to tell.

Updates and Engagement

Give updates about the campaign to donors. Update the campaign page and blog to keep donors informed on how the patient is doing. As the campaign progresses it is important to continue to engage not only donors but to let everyone know how the campaign is going.

People want to see you succeed. Always mention the mission of the campaign and any progress on the campaign or money raised. Donors did not give so you have money, they gave to help support the patient, a person, a little girl. Don't take credit for their work.

Most people are generally interested in learning and growing. Try to make your campaign mean something to them. Show how it will directly impact their life. Keep updating once or twice a day, especially between 7:00 am and 9:00 am EST and again between 6:30pm- 9:30 pm EST since these are the times people are most likely to use social media. With regular updates, the audience feels connected, and they often donate again. They think, "I have an extra $5 today." When they comment on the campaign, you can see their connection to the cause and so can other potential donors. You have to post every day. If you don't post, they can't share. Provide status updates on the health of the patient.

Talk about any media hits. For example, in an update, link a blog post about your campaign and at people to share it. Or provide photo updates: "Here is a picture of our daughter before she was sick, and here is a picture of her in treatment now." And whenever you can, let the patient speak for him- or herself.

In medical campaigns, inform and engage the audience on the disease state. Explain how it is similar to or different from other diseases. If the patient has a rare disease, talk about current work on rare diseases in an update. Discuss the latest technology of genome sequencing and its potential for rare diseases.

Always remember to thank everyone for their involvement, support, shares, and feedback for the campaign. Have a specific person in charge of social media for your campaign if possible so you don't get overwhelmed.

Mission

Never mention money without mentioning the mission. Avoid saying, "we raised," this amount of money. Instead always give the donors the credit by saying, "You've donated." Allow specific facts to make the story of living with the disease come to life. For example, mention the patient having their blood drawn many times in a day.

The goal is to make a connection with the audience and create interest through updates. "Every day I include a photo and video," said Susanne Shaw, Rett Cavan's aunt and campaign organizer. As long as it is a crisp video with good lighting, it does not need to be professional. Many apps for creating movies and videos are free; research what is available and become proficient with the technology.

Ask people to share every couple of days. "On Instagram, hashtags are used for 30 seconds to a minute. You can change hashtags on existing posts multiple times each day, and each time you do, it will refresh. I linked my Instagram account to the GoFundMe page. Many donations are only $5. That is ok because $5.00, but $5.00 really adds up when many people donate that amount," advises Susanne.

Work together toward a common goal. Finally, take advantage of other campaigns with similar values by engaging in cross promotion. This way both campaigns have twice as many contacts.

Updates Checklist:

- ☑ Always state the mission
- ☑ The more stories you share, the more traffic you will get. Tweet and post on Facebook, Instagram and any social media sites you are active.
- ☑ More content equates to more donations. Provide status updates on the patient's health - the patient's perspective is important to share.
- ☑ Provide a status on the campaign.
- ☑ Talk about media coverage.
- ☑ Share new photos.
- ☑ Updated videos.
- ☑ Provide updates on disease state information
- ☑ Recognize those who have donated.

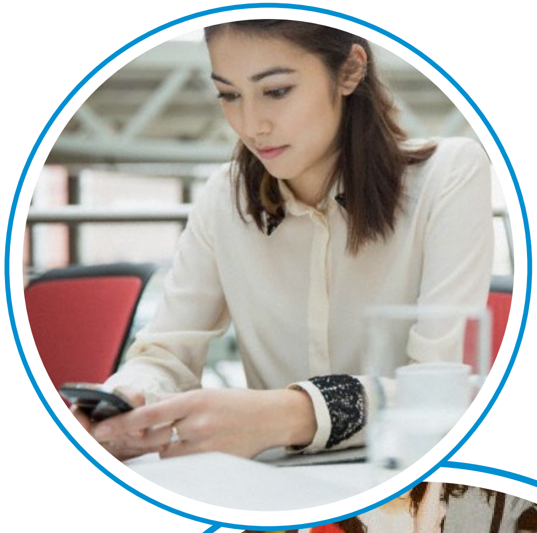

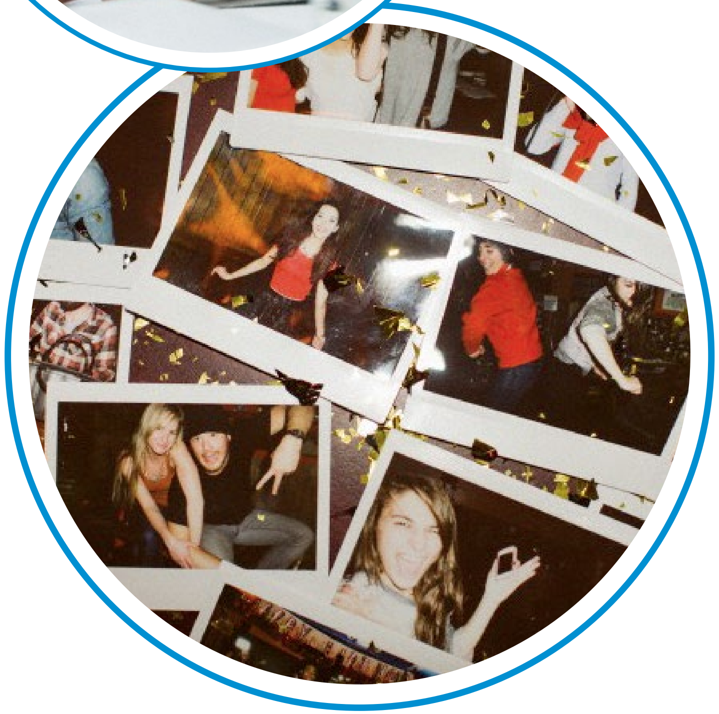

Timeline

Having an end date to the campaign helps create urgency. If the money is for an upcoming clinical trial or medical procedure, tell people so they understand why you need the money soon.

Early momentum is key. According to Kickstarter, if a campaign is not funded within 45 days, its chances of getting funded decrease significantly. However, sometimes it makes sense to set a longer time period; a lung transplant project researcher raised $100 from each of 20 donors over the course of four months. It's hard to know the exact, appropriate time just remember that early momentum and an established end date are important.

Maintain the excitement. Most campaigns last 30-60 days and most donations occur during the first 30 days of properly planned campaigns. You want to maintain the buzz about your campaign. There has to be a deadline to create the urgency. One of your most important updates will be reminding people about the timeline. Toward the end of the campaign, you need their help the most to reach the goal.

Timeline Checklist:

1. Have a start and end date.
2. Create urgency.
3. Reinforce the message.

Offline Fundraising Efforts

Publicize all offline fundraising efforts on your campaign page and social media. Publicizing your offline fundraising demonstrates that you are doing everything you can to reach your goal.

For example, Roger and Jeneva Stone quickly reached their crowdfunding goal, but Jeneva also had an idea for creating holiday cards that showed people how to donate to their campaign with a link.

Team inquiry. Ask your team members to reach out to all of their contacts and organizations from daily life. Let them do the initial inquiry and don't go along to the meeting yourself unless your friend recommends it. This allows people to say no without feeling uncomfortable. This also saves you from unnecessary stress.

Speak at events. Research and reach out to organizations that offer "lunch and learn" kinds of events. Presenting your campaign to a group is a great offline opportunity to talk about your cause, raise awareness, broaden the reach of your network and gain new donors. One campaign organizer raising money for a research project had an offer from a friend to talk at McKinsey. She discussed her research project and everyone was excited about it.

Zsusanna Darvai took many opportunities to speak about the campaign for her son Balazs. One of Zsuzsanna Darvai's successful crowdfunding moments came when her older son's martial arts teacher heard about her effort and offered to do a kick-a-thon, their usual fundraising event, to benefit the family cause. Events like kick-a-thons, walk-a-thons, and read-a-thons include everyone in a particular organization like a gym or library and expand your circles. If you go this route, remember to thank the organization and its members on your site.

Zsuzsanna also approached smaller, local restaurants who were unable to donate money, but offered to provide food at an event. Many restaurants will give gift cards and related "prizes" for your cause.

Create a Flyer. Susanne Shaw used similar methods for her nephew's campaign. She brought a flyer she created to local businesses to more effectively solicit donations. She also asked to post her flyer. Eventually she was able to secure donations of goods or services from local restaurants, bands and even a beautician who donated the money from 12 hours of her work. What could you include in a flyer to post at local businesses, the library, and the community center? Briefly summarize the campaign, show a picture of the person who is benefitting, and provide your link.

Advice and Feedback

What do you think? Successful crowdfunding campaign organizers agree that advice and feedback can be invaluable for your crowdfunding campaign. Talk to both people who had successful crowdfunding campaigns and those whose campaigns didn't meet their goal to better plan your own campaign.

Just ask. The biggest hurdle for most people beginning their first crowdfunding endeavor is to let go of inhibitions and ask for help. However, the worst case scenario is that people do not contribute to the campaign. That's it, so don't take it personally. It's not a comment on the value of the campaign.

The Final Push to the Campaign Goal

Rare Genomics Institute

THE FINAL PUSH TO THE CAMPAIGN GOAL

Techniques and Metrics

If you are $2000 away from the goal, let everyone know you are almost there. This is not a huge amount to be funded by a large group. Keep people engaged by explaining your story and mission again and thank everyone for their contributions so far. Then let them know that you are almost at your goal but need them for one final push. Many successful campaigns get repeat donors.

Make sure you are monitoring these aspects of your campaign:

- Conversion: how many people who see also donate?
- How many shares are you getting per week? Per month?
- How many likes on Facebook?
- How many unique visitors?
- What are the page views per visitor?
- How many new campaign site users?
- How many clicks?
- What is the total time spent on site by visitors?
- Total time spent per user?
- What is the frequency of visits? How do visitors participate?
- What is the user generated content?

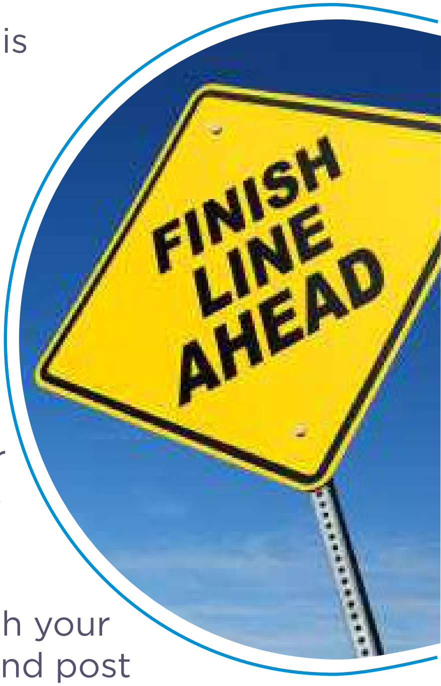

Stay involved. While your campaign is active, and especially as you head to the finish line, you will have to be involved daily. Donors expect transparency, so share information with them to cultivate partnerships. Acknowledge the active roles inherent to crowdfunding by saying "please help fundraise," not "please donate." A fundraiser will be actively involved, emailing all of their contacts, and this is a more effective strategy.

Say thank you-again. When you finish your campaign, thank all of your donors and post the final update and your results. Let everyone including your media contacts know that you reached or exceed your goal and that you could not have done it without them. This will further highlight your cause and raise awareness.

Culture Change in Science

Crowdfunding has prompted a culture change in the scientific community. In academia, funding is the single biggest issue. A big asset crowdfunding offers is the ability to share the results with the donors. Crowdfunding supports ideas that would otherwise go unfunded. Researchers have confirmed that interaction with donors can change their perspective on how to conduct research.

Role models. Researchers spend too much valuable time writing grants. This is the crowdfunding advantage: it allows researchers the resources they need to do the important work. Among the best ways to increase interest in and awareness of science is to foster the work of researchers to serve as role models. This helps young people to see the role science can play in their lives.

Researchers should think of potential donors as members of their virtual lab. They have joined their team. They means they must keep the lines of communication open. With this shift in the donor base, you don't have to donate $100,000 to be connected to the project. The researcher should use a conversational tone to give updates and information; this deepens engagement.

Final Thoughts

Throughout this ebook, you have learned tools and tips from the experts on how to have a successful crowdfunding campaign. Planning is key. Build your team and prepare your messaging early. Ask for feedback and thank everyone that supported and helped your campaign. Crowdfunding offers a unique opportunity to engage your network to support your cause.

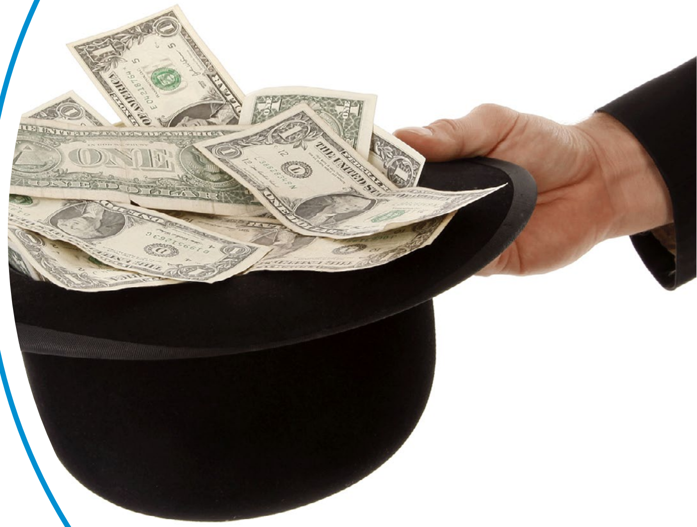

Appendix

Rare
Genomics
Institute

APPENDIX

With more than 500 crowdfunding platforms, we will focus on the top sites for personal medical causes and those that specialize in raising money for research.

Crowdfunding Platform	All or Nothing	Keep it All	Website fee	Processing Fee	Support
CauseVox		✔	2.5-5% based on plan	2.9% + $0.30 fee per donation	Yes
CrowdRise		✔	3-5% based on plan	2.9% + $0.30 fee per donation	Yes
Experiment	✔		5%	3% if the goal is reached	Yes
OzCrowd	✔	✔	2.9%	2.5% + $0.30 fee per donation	Yes
Piggybackr		✔	Up to 5% based on plan	2.9% + $0.30 fee per donation	Yes
GoGetFunding		✔	4%	2.9% + $0.30 fee per donation	Yes
**Causewish**		✔	4%	2.9% + $0.30 fee per donation	Yes

Crowdfunding Platform	All or Nothing	Keep it All	Website fee	Processing Fee	Support
Medstartr	✔	✔	5% All or Nothing<br>8% Keep It All	2.9% + $0.30 fee per donation	Yes
YouCaring		✔	no	2.9% +$0.30 fee per donation	Yes
GiveForward		✔	5%	2.9% +$.50 fee per transaction	Yes
Indiegogo	✔	✔	4% if goal attained	3-5%	Yes
GiveCorps		✔	3%		Yes
GoFundMe		✔	5%	3%	Yes
Fundly		✔	4.9%	2.9% +$0.30 per transaction	Yes
Fundrazr		✔	5%	2.9%+ $0.30 per transaction	Yes
RocketHub	✔	✔	4% if goal attained<br>8% if goal not attained	4%	Yes

Contributors

Rare
Genomics
Institute

CONTRIBUTORS

Denny Luan, co-founder
Experiment

Molly Lindquist, founder
Consano

Rob Wu, founder
CauseVox

Andrea Lo, co-founder and CEO
Piggybackr

Nick Karolidis, Director
OzCrowd

Sandip Sekhon, CEO & Founder
GoGetFunding and CauseWish

Adam Griff, COO
Medstartr

Annette Hayswirth, Manager
CrowdRise

Luke Miner, co-founder
YouCaring

Ethan Austin, co-founder
GiveForward

Devin Thorpe
Crowdfunding expert and author of *Crowdfunding for Social Good, Financing Your Mark On The World*

Breanna DiGiammarino, Head of Causes
Indiegogo

Elizabeth Iorns, co-founder
Science Exchange

Nick Sireau, Chairman and co-founder
Findacure

Jamie McDonald, founder
GiveCorps

SUCCESSFUL CAMPAIGN CONTRIBUTORS

Jeneva Stone, mother of Robert
Rare Genomics Institute

Zsuzsanna Darvai, mother of Balazs
Rare Genomics Institute

Ignacio Garcia, father of Joaking
Rare Genomics Institute

Sam De Brouwer, co-founder
Scanadu Scout campaign on Indiegogo

Glenn O'Neill, father of Eliza
Saving Eliza campaign on GoFundMe

Susanne Shaw- aunt and organizer for Rett
Rett's Medical Relief Fund on GoFundMe

Dr Kimmie Ng, Assistant Professor of Medicine
Dana Farber Cancer Institute
Role of Vitamin D Supplementation in Colorectal Cancer on Consano

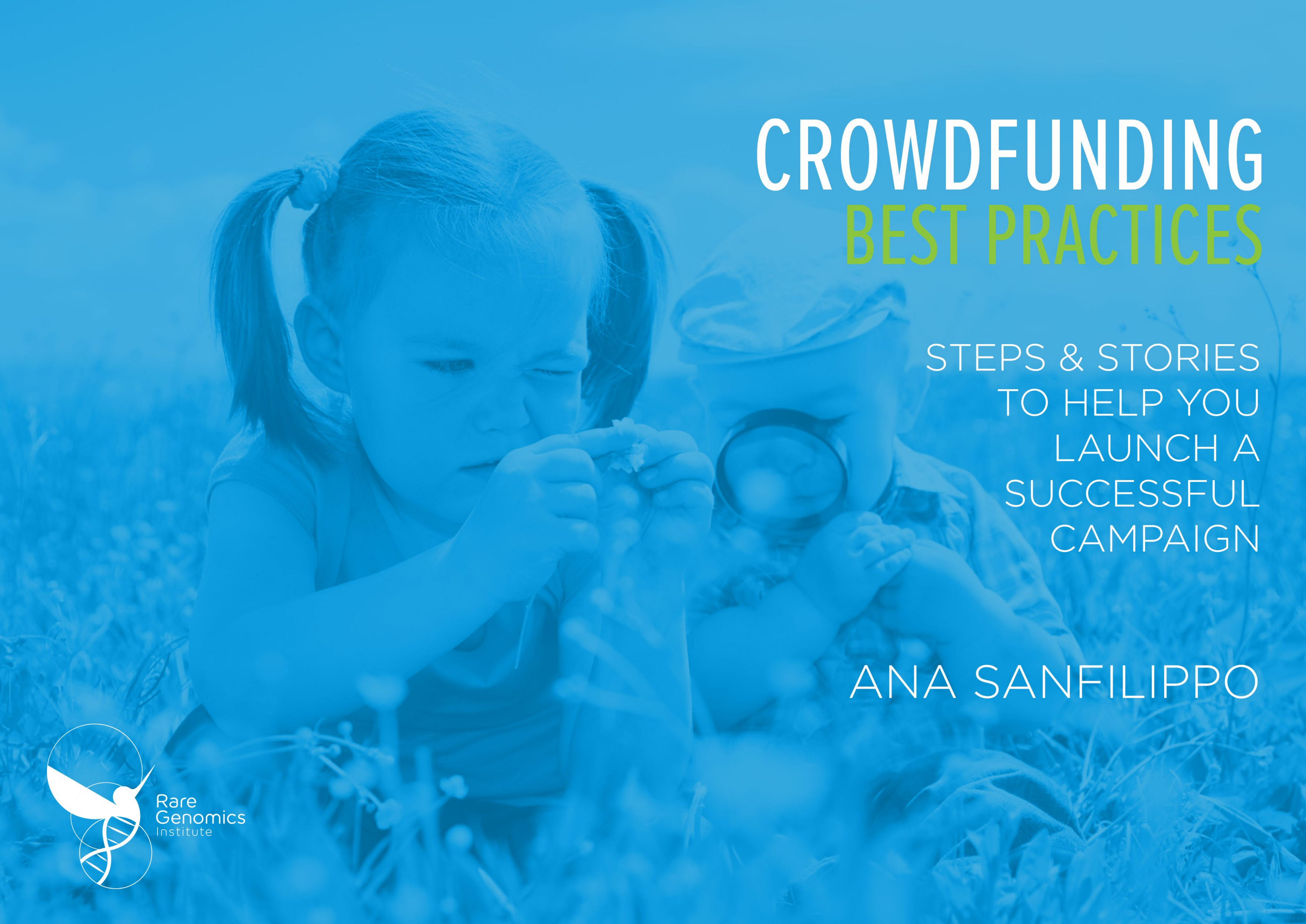

CROWDFUNDING
BEST PRACTICES

STEPS & STORIES
TO HELP YOU
LAUNCH A
SUCCESSFUL
CAMPAIGN

ANA SANFILIPPO

Rare
Genomics
Institute
