# Supplementary material for: Engaging a Community for Rare Genetic Disease: Best Practices and Education From Individual Crowdfunding Campaigns
Source: Interact J Med Res. 2018 Feb 5;7(1):e3. doi: 10.2196/ijmr.7176 (PMC5818677; doi:10.2196/ijmr.7176)
Supplement: Multimedia Appendix 6 [file ijmr_v7i1e3_app6.pdf]

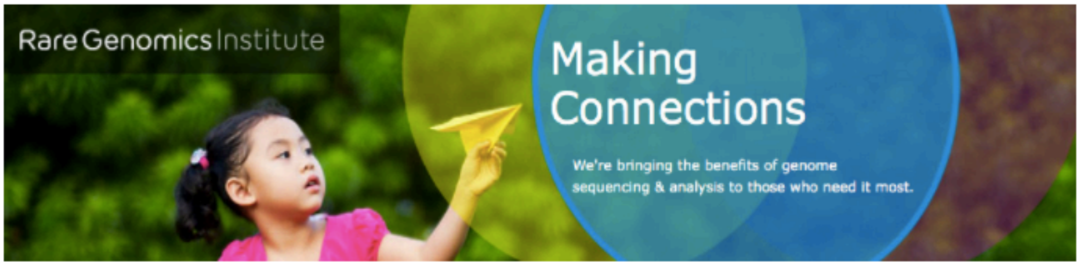

Donor

*** 2. How familiar were you with genomics? Where 0 is not knowing anything about genomics and 10 is being an expert**

0	1	2	3	4	5	6	7	8	9	10
○	○	○	○	○	○	○	○	○	○	○

*** 3. What material did you receive / read / watch from this campaign? (check all that apply)**

- ☐ read the campaign summary
- ☐ looked at other similar campaigns
- ☐ watched TedxMidAtlantic video
- ☐ read Genomics 101
- ☐ read ebook(s)
- ☐ other (please specify)

*** 4. After donating to this campaign, how knowledgeable are you about genomics. Where 0 is not knowing anything about genomics and 10 is being an expert.**

0	1	2	3	4	5	6	7	8	9	10
○	○	○	○	○	○	○	○	○	○	○

5. Which campaign did you donate to?

- ○ Ali
- ○ Charlie
- ○ Cure Seeker
- ○ Fedor
- ○ Javier
- ○ Kendra
- ○ Ria
- ○ Stryder
- ○ Tommy

*** 6. What is your relationship with the patient / family? (check all that apply)**

- ☐ family or relative
- ☐ co-worker
- ☐ friend
- ☐ friend-of-friend
- ☐ none
- ☐ other (please specify)

7. How did you hear about this campaign? (check all that apply)

- [ ] word-of-mouth
- [ ] email
- [ ] phone call
- [ ] social media
- [ ] newspaper / print
- [ ] TV / radio
- [ ] other (please specify)

8. If you selected social media in question 7, which social media network? (please select all that apply)

- [ ] Facebook
- [ ] Twitter
- [ ] Instagram
- [ ] LinkedIn
- [ ] Google+
- [ ] Tumblr
- [ ] Flickr
- [ ] Pinterest
- [ ] Path
- [ ] Vine
- [ ] YouTube
- [ ] Reddit
- [ ] other (please specify)

*** 9. How do you plan to share this campaign with others? (check all that apply)**

- [ ] word-of-mouth
- [ ] social media
- [ ] email
- [ ] letter
- [ ] phone
- [ ] none
- [ ] other (please specify)

Prev Next

Donor

* **10. I understand what the funds for this project go towards.**

strongly agree	agree	neither agree or disagree	disagree	strongly disagree
○	○	○	○	○

* **11. How many times have you donated to a crowdfunding campaign?**
(e.g. Kickstarter, CrowdRise, Indiegogo, YouCaring, GoFundMe, Razoo, GiveForward, etc)

- ○ never
- ○ once or twice
- ○ 3-5 times
- ○ 6-10 times
- ○ more than 10

* **12. Which of the following methods do you feel are the most effective ways to learn about genetic sequencing and genomics? (check all that apply)**

- ☐ webinars
- ☐ videos
- ☐ e-newsletters
- ☐ case studies
- ☐ fact sheets/ reports/ technical papers
- ☐ other (please specify)

* **13. After donating to this campaign how interested are you in learning about genomics and personalized medicine?**

extremely interested	interested	neither interested or disinterested	somewhat interested	not at all interested
○	○	○	○	○

* **14. How much of an impact do you feel your donation makes?**

a great deal	a lot	a moderate amount	a little	not at all
○	○	○	○	○

* **15. How important is it for you to fund crowdfunding projects that have already received substantial donations from others?**

very important	important	moderately important	of little importance	unimportant
○	○	○	○	○

* **16. How important is it to fund projects that are close to meeting their fundraising goal?**

very important	important	moderately important	of little importance	unimportant
○	○	○	○	○

* **17. How important is it to you to fund projects that are close to the campaign ending or a deadline?**

very important	important	moderately important	of little importance	unimportant
○	○	○	○	○

*** 18. What is your gender?**

- ◯ Female
- ◯ Male
- ◯ prefer not to answer

*** 19. What is your age?**

- ◯ 18 to 24
- ◯ 25 to 34
- ◯ 35 to 44
- ◯ 45 to 54
- ◯ 55 to 64
- ◯ 65 to 74
- ◯ 75 or older

*** 20. What is the highest level of school you have completed or the highest degree you have received?**

- ◯ Less than high school degree
- ◯ High school degree or equivalent (e.g., GED)
- ◯ Some college but no degree
- ◯ Associate degree
- ◯ Bachelor degree
- ◯ Graduate degree

Prev Done
